# Stopping, starting, and sustaining HIV antiretroviral therapy: a mixed-methods exploration among African American/Black and Latino long-term survivors of HIV in an urban context

**DOI:** 10.1186/s12889-021-10464-x

**Published:** 2021-02-27

**Authors:** Marya Gwadz, Charles M. Cleland, Robert Freeman, Leo Wilton, Linda M. Collins, Robert L. Hawkins, Amanda S. Ritchie, Noelle R. Leonard, Danielle F. Jonas, Alexis Korman, Sabrina Cluesman, Ning He, Dawa Sherpa

**Affiliations:** 1 Center for Drug Use and HIV/HCV Research (CDUHR), New York University School of Global Public Health, New York, NY USA; 2 New York University Silver School of Social Work, New York, NY USA; 3 Division of Biostatistics, Department of Population Health, New York University School of Medicine, New York, NY USA; 4 Independent Consultant, Brooklyn, NY USA; 5 Department of Human Development, State University of New York at Binghamton, Binghamton, NY USA; 6 Faculty of Humanities, University of Johannesburg, Auckland Park 2006, Johannesburg, South Africa; 7 Methodology Center and Department of Human Development and Family Studies, Pennsylvania State University, State College, PA USA; 8 New York University School of Medicine, New York, NY USA

**Keywords:** HIV survivorship, HIV antiretroviral therapy, Non-persistence, Initiation, Disparities, Resilience, Race/ethnicity, Substance use, Poverty, Mixed methods

## Abstract

**Background:**

Although periods of HIV antiretroviral therapy (ART) discontinuation have deleterious health effects, ART is not always sustained. Yet, little is known about factors that contribute to such ART non-persistence among long-term HIV survivors. The present study applied a convergent parallel mixed-methods design to explore the phenomena of stopping/starting and sustaining ART, focusing on low-socioeconomic status African American or Black and Latino persons living with HIV (PLWH) who face the greatest challenges.

**Methods:**

Participants (*N* = 512) had poor engagement in HIV care and detectable HIV viral load. All received structured assessments and *N* = 48 were randomly selected for in-depth interviews. Quantitative analysis using negative binomial regression uncovered associations among multi-level factors and the number of times ART was stopped/started and the longest duration of sustained ART. Qualitative data were analyzed using a directed content analysis approach and results were integrated.

**Results:**

Participants were diagnosed 18.2 years ago on average (SD = 8.6), started ART a median five times (Q1 = 3, Q3 = 10), and the median longest duration of sustained ART was 18 months (Q1 = 6, Q3 = 36). Factors associated with higher rates of stops/starts were male sex, transgender identity, cannabis use at moderate-to-high-risk levels, and ART- and care-related stigma. Factors associated with lower rates of stops/starts were older age, more years since diagnosis, motivation for care, and lifetime injection drug use (IDU). Factors associated with longer durations of sustained ART were Latino/Hispanic ethnicity, motivation for ART and care, and recent IDU. Factors associated with a shorter duration were African American/Black race, alcohol use at moderate-to-high-risk levels, and social support. Qualitative results uncovered a convergence of intersecting risk factors for stopping/starting ART and challenges inherent in managing HIV over decades in the context of poverty. These included unstable housing, which contributed to social isolation, mental health distress, and substance use concerns, the latter prompting selling (“diverting”) ART. Primarily complementary quantitative and qualitative findings described mechanisms by which risk/protective factors operated and ways PLWH successfully restart and/or sustain ART.

**Conclusions:**

The field focuses substantially on ART adherence, but greater attention to reducing the frequency of ART non-persistence is needed, along with creating social/structural conditions favorable for sustained ART.

**Supplementary Information:**

The online version contains supplementary material available at 10.1186/s12889-021-10464-x.

## Background

In the third decade of the HIV epidemic, there is growing optimism, particularly in higher-resource settings such as the United States, about the possibility of reaching the 90–90-90 goals set by the World Health Organization, in which 90% of those with HIV infection will be diagnosed, 90% of these will receive HIV antiretroviral therapy (ART), and 90% of these will achieve HIV viral suppression [1]. Indeed, reaching these 90–90-90 targets is critical to achieving the larger public health objective of ending HIV transmission, referred to as “ending the HIV epidemic” [2]. To accomplish these public health goals, it is necessary for persons living with HIV (PLWH) to engage in regular HIV primary care [3, 4]. Further, it is recommended that PLWH initiate ART as soon as possible after diagnosis and take ART with high levels of adherence consistently over time (that is, take ART every day, exactly as prescribed) in order to achieve and sustain HIV viral suppression [5–8]. Yet, while the majority of PLWH in the United States engage in HIV care and initiate ART in a timely fashion after diagnosis [9], it is clear that in many cases ART is not continuously sustained [10]. Indeed, in a review article, Bae and colleagues [10] highlighted that continuous treatment with ART over time, called ART persistence, is an under-recognized aspect of the HIV care continuum [10]. Yet, ART *non*-persistence; that is, not taking ART for at least two consecutive days related to a self-initiated decision or external factor [11], has very serious deleterious effects on health. Those who do not continuously persist on ART have higher morbidity and mortality rates compared to those who persist [12]. Moreover, the frequency and duration of non-persistent ART episodes appear to be better predictors of poor clinical outcomes in PLWH than ART non-adherence; that is, missing doses of ART while staying on an ART regimen [10, 11]. Yet, in contrast to the large literature on ART non-adherence [11], substantially less is known about ART non-persistence.

### Patterns of ART non-persistence

Despite the importance of sustained ART use, non-persistence is common, particularly among populations of PLWH in high-risk contexts. For example, in a national study of ART discontinuation among low-income PLWH in the United States, a substantial proportion (44%) did not reinitiate ART over an 18 month period, and among those who did reinitiate ART, the median time between discontinuation and re-initiation was 8.2 months [13]. In a study of persons who inject drugs in Baltimore, 78% of individuals had at least one self-initiated treatment discontinuation episode, and a substantial proportion (20%) never re-initiated ART [14]. In contrast, only 5.6% discontinued ART in a nationally representative, cross-sectional sample of adult PLWH who were not embedded in high-risk contexts (i.e., they were receiving HIV care, mostly male, self-identified as heterosexual, and had some college education or more, an income above the poverty level, and insurance for the entire previous 12 months) [15]. Existing studies suggest that ART non-persistence can take various forms; for example, PLWH can stop ART for short periods of time, referred to as “drug holidays” [11] or brief unstructured anti-retroviral treatment interruptions [16], and then re-start. While stopping ART is certainly not generally recommended, re-starting medication may be a sign of resilience [17]. Alternately, PLWH can discontinue and remain off ART for substantial periods of time, raising the risk of adverse effects on immune functioning and general health [13, 14, 16].

### Multi-level factors associated with ART non-persistence

Existing research on factors associated with ART non-persistence are consistent with the literature on known barriers to other HIV-related outcomes such as engagement in HIV care and ART adherence. For example, younger PLWH, women, African American/Black and Latino individuals, and those with higher HIV viral load, hepatitis C virus, lack of medical coverage, and fewer years living with HIV and/or less time on ART, have been more likely to discontinue ART than their peers who do not face these barriers [10, 14, 15, 18–20]. Further, heavy or hazardous substance use, including related to injection drug use (IDU), mental health distress, particularly depression, and incarceration contribute to ART non-persistence [14, 15, 19, 20]. Aspects of health care settings contribute to ART non-persistence, including fragmented services and less frequent monitoring, particularly when ART is first initiated [10]; in contrast, engagement in any type of health care predicts ART re-initiation among those who discontinue [13]. Moreover, low socioeconomic status (SES) and chronic poverty are strongly associated with ART non-persistence [10, 14, 15, 18–20]. Yet, compared to the very large literature on ART adherence, substantially less is known about the specific mechanisms by which these types of factors cause or contribute to ART non-persistence, particularly past one- or two-year time horizons [4], or about factors that potentially promote re-initiation of ART and/or sustained ART use, including in times of hardship, when the risk for discontinuation may be high. The present study addresses this gap in the literature.

### Objectives of the present study

The present study takes a convergent parallel mixed-methods approach [21] to contribute to the literature on ART non-persistence, focusing on African American/Black and Latino PLWH from low-SES backgrounds who have lived with HIV for a decade or longer and who have taken ART in the past. We focus on African American/Black and Latino PLWH from low-SES backgrounds because this subgroup experiences the greatest barriers to engagement along the HIV care continuum and to favorable HIV health outcomes including longevity, compared to their high-SES and White peers [22, 23]. Within the domain of ART non-persistence, we were interested both in the phenomena of stopping and starting ART as well as sustaining ART. Indeed, little is known about which specific factors contribute to PLWH stopping ART and what prompts them to restart ART, and even less is known about the factors that contribute to PLWH sustaining ART – from the perspectives of this subpopulation of PLWH. The present study is guided by the theory of triadic influence, a social-cognitive theory that highlights the importance of individual-, social-, and structural-level streams of influence on health behavior [24], as well as self-determination theory, which emphasizes how autonomy, competence, and relatedness drive self-motivated action [25, 26]. Further, consistent with the transformative mixed-methods paradigm, we draw on critical race theory [27] to “center” the experiences of the study population in the research design and analyses, and underscore the importance of structural causes of poor health outcomes, including structural racism; that is, the macro-level systems that reinforce inequities among racial/ethnic groups. We refer to this as the integrated theoretical model. The primary objective of the present study was to identify and understand multi-level factors that contribute to African American/Black and Latino PLWH stopping and starting ART and those associated with sustaining ART, including during times of difficulty when the risk for ART discontinuation may be high. We focused on risk factors as well as protective factors and evidence of resilience.

## Methods

### Description of the present study

As noted above, the present cross-sectional study uses a convergent parallel mixed-methods design. In this approach, the quantitative and qualitative data are collected and analyzed independently, the methods are weighed equally, and results are interpreted together [21]. Further, the study draws on the transformative paradigm in that it centers the experiences of marginalized populations, and considers power differentials that have led to marginalization [21]. This mixed-methods approach was selected to uncover and describe agreements and contradictions between quantitative and qualitative findings to thereby enrich study findings [21]. Guided by the integrated theoretical model described above, and using quantitative data, we examined what socio-demographic and background, HIV health-related, and individual-, social-, and structural-level factors were associated with two outcomes of interest: 1) the number of times participants stopped and started ART in the period since they first initiated ART (referred to below as “ART starts”) and 2) the longest duration of sustained ART since participants first initiated ART. Taking into account the complexity of managing ART, we assumed that a lower number was generally preferable to a higher number of ART starts [28]. Because we targeted PLWH who had lived with HIV for a decade or longer, we focused mainly on lifetime or longstanding potential predictors of these outcomes, such as adverse childhood experiences, the location where HIV was first diagnosed (e.g., prison, hospital), lifetime incarceration, and substance use treatment, along with a select set of more recent attitudes, substance use patterns, and social (e.g., stigma) and structural (e.g., perceived barriers to HIV care, housing status) factors [29, 30]. Because the study was exploratory, we do not present formal hypotheses, but each of the indices we examined was plausibly related to the outcome variables based on prior research [10, 13, 15]. We predicted that ART starts and sustaining ART were related behaviors and thus factors associated with ART starts would be similar to those associated with the longest duration of sustained ART. Qualitative research questions included: What are PLWH’s perspectives on why they stop taking ART, what prompts them to restart ART, and how and why do they sustain ART over time, including during times of adversity when the risk for ART discontinuation may be high? How do these factors operate in isolation and in combination with each other? We also examined whether there were important emergent factors that drive this subpopulation of PLWH to stop, restart, or sustain ART not well-described in the literature to date.

The present study drew on two sources of data drawn from a larger intervention optimization research project using the multiphase optimization strategy (MOST) conducted between April 2017 and November 2019 [31, 32]: quantitative interview data from a baseline assessment and semi-structured in-depth interview data. MOST is a pioneering engineering-inspired framework for testing individual intervention components before combining them into an optimized multi-component intervention [32, 33]. The larger study was a factorial experiment designed to test the efficacy of five separate culturally salient intervention components (patient navigation, counseling sessions, pre-adherence habit formation, peer mentorship, and support groups). Conducted in New York City (NYC), it focused on African American/Black and Latino PLWH from low-SES backgrounds who were poorly engaged along the HIV care continuum—specifically, those who did not engage in HIV care at recommended levels and who evidenced both poor adherence to ART and detectable HIV viral load. Participants gave signed informed consent for study activities, and the consent process included information about compensation levels. Compensation was designed to be comparable to amounts participants could earn for their time engaging in other similar activities, while not being coercive [34]. The study was approved by the Institutional Review Board at the New York University Langone Medical Center.

### The local context

NYC is a location with a large and mature HIV epidemic of approximately 127,000 PLWH, more than 75% of whom are African American/Black and Latino [35]. HIV prevalence and HIV-related death rates are highly concentrated in the highest-poverty neighborhoods of NYC, which are predominantly African American/Black and Latino [35], highlighting the intersection of race/ethnicity and SES among PLWH. NYC provides a large network of HIV care facilities and related support services [36] and PLWH have access to HIV care and ART at low or no cost, regardless of immigration status [36]. PLWH in NYC are typically eligible for Medicaid, a publicly funded federal insurance assistance program for low-income individuals [37, 38]. NYC has achieved somewhat higher rates of engagement along the HIV care continuum compared to national figures (e.g., 77% of all PLWH in NYC are virally suppressed) [35]. Nonetheless, NYC also evidences serious racial/ethnic disparities in engagement along the HIV care continuum. Racial/ethnic disparities in engagement in care, ART initiation, and viral suppression are similar to national patterns, where African American/Black and Latino PLWH in low-SES locations show lower rates of engagement along the HIV care continuum compared to White PLWH in higher SES locations [22, 39].

### Eligibility criteria

The larger study’s inclusion criteria were 1) age 18–65 years; 2) African American or Black race or Latino or Hispanic ethnicity (we use the term Latino throughout the paper for parsimony); 3) HIV diagnosed for at least 6 months; 4) ART adherence less than 50% in the past 6 weeks and detectable HIV viral load based on a laboratory report; 5) sub-optimal engagement in HIV care (operationalized as less than one visit in every 4 month period in the past year [two of them at least 90 days apart], pro-rated for those diagnosed less than a year ago) *or* > two missed visits without prior cancellation in the past year [3]; 6) resides in the NYC metropolitan area; 7) not planning to leave the NYC metropolitan area in the next year; 8) not actively psychotic based on a screening instrument; 9) able to conduct research activities in English or Spanish; 10) willing to provide a blood specimen at screening (to assess HIV viral load); and 11) willing to be randomly assigned to 1–5 intervention components that focus on HIV care and ART initiation/adherence.

### Recruitment into the larger study

Recruitment of PLWH with poor engagement along the HIV care continuum is challenging [40–42]. Participants were recruited using a hybrid sampling strategy comprised of peer-to-peer recruitment, direct recruitment by study staff members in HIV service, HIV housing, and other community-based organizations, and advertisements in a local free newspaper. Peer recruitment was the primary sampling approach. Peer recruitment was tracked with a coupon system that linked the recruiter to the recruit, and recruiters received modest compensation for recruitment ($15/recruit). Most enrolled participants were recruited by peers (75%); 9% were recruited through newspaper ads, and 16% through other means.

### Procedures

#### First-stage screening (by phone)

Potential participants contacted the study directly, generally by phone, and usually with a coded recruitment coupon provided to them by a recruiter. Verbal informed consent was elicited following an IRB-approved script. Participants then engaged in a brief structured screening interview (~ 10 min) administered by a staff member to determine preliminary eligibility. The screening interview assessed the following inclusion criteria: age, race/ethnicity, residence in NYC metropolitan area, HIV diagnosis, adherence patterns, and HIV care engagement patterns. No compensation was provided for this brief phone screening. Those found preliminarily eligible at the first-stage screening proceeded to the second stage.

#### Second-stage screening (in person)

Participants provided signed informed consent followed by a review of medical documentation to confirm HIV status. Then, participants were escorted by a study staff member to a local commercial laboratory for HIV viral load testing. Participants were instructed on peer recruitment procedures and received recruitment coupons at this time. Participants were compensated $15 for the screening interview and $15 for completing the blood specimen draw, along with funds for local round-trip public transportation.

#### Enrollment, baseline assessment, randomization, and in-depth interview

HIV viral load results were typically available from the laboratory within 1–4 days. Those found eligible at this stage based on HIV viral load results were contacted by phone and invited to enroll in the larger study. After providing signed informed consent, participants completed a structured baseline assessment battery lasting 60–90 min. The baseline assessment was conducted on the REDCap platform and was comprised of computer-assisted personal interviewing (CAPI) and audio, computer-assisted self-interviewing (ACASI) formats. After completing the baseline assessment, participants were randomly assigned to an intervention condition. Consistent with the MOST framework, the study used a fractional factorial design, and participants were randomly assigned to one of 16 different conditions, where each condition was comprised of a different combination of the five intervention components [31]. Most conditions were comprised of 2–4 components. Then, a total of 2–4 participants from each of the 16 conditions were randomly selected for two qualitative, semi-structured, in-depth interviews, one early in the study (within 5–7 months of enrollment) and another at study completion. The first qualitative interview focused mainly on participants’ general experiences living with HIV and with engagement along the HIV care continuum, and the second on perspectives on the larger study’s activities. The in-depth interviews were audio-recorded and professionally transcribed verbatim. Individuals were compensated $25 for each assessment, along with funds for round-trip local transportation. In-person study activities took place in confidential offices at a project field site in lower Manhattan in NYC. The present study uses quantitative baseline data from all participants (*N* = 512) and qualitative data from participants who engaged in the first in-depth interview (*N* = 48).

### Measures

The structured instruments that made up the baseline assessment battery have been found reliable and valid with similar populations in high-risk contexts [43]. Cronbach’s α for scales is reported where appropriate.

#### Socio-demographic and background characteristics

Age, sex assigned at birth, gender identity, sexual minority status (identifies as gay, lesbian, bisexual, queer, or other non-heterosexual identity), race/ethnicity, education level (high school or equivalency or higher, yes/no), lifetime history of homelessness (yes/no), lifetime history of incarceration (yes/no), and the number of times incarcerated were assessed with structured instruments developed for populations in high-risk contexts [43].

#### HIV history and health

We used a version of the HIV Cost and Services Utilization Study (HCSUS) [44] instrument to assess years since first HIV diagnosis; years since first initiated ART; number of months since last ART dose (if not on ART at screening); whether perinatally infected (yes/no); whether diagnosed in jail/prison, a hospital emergency department, or hospital inpatient unit (yes/no); number of co-occurring physical and mental health conditions diagnosed with over the lifetime (e.g., asthma, hepatitis C virus, diabetes, heart disease, range 0–21); whether covered by a health insurance plan (yes/no); number of times stopped and started ART; and the longest amount of time ever on ART (in months).

Reasons for stopping HIV medication were assessed with a 13-item scale assessing factors such as “you did not want side effects,” “you decided to stop because your CD4 (a marker of immune health where higher values are better than lower) and viral load numbers (a marker of HIV disease where lower values are better than higher) have been good,” and “you do not want anyone to find out that you are HIV positive” (yes/no) [45].

#### Adverse Childhood Experiences Scale- revised (ACES-R)

The ACES-R assesses early life experiences such as physical abuse, neglect, and sexual abuse [46, 47]. We used a version of the ACES developed by Finkelhor and colleagues [29] that included additional widely recognized childhood adversities: peer victimization, peer isolation/rejection, and community violence exposure (e.g., Did you live for two or more years in a neighborhood that was dangerous, or where you saw people being assaulted?). The ACES-R scale has 14 items that are answered on a yes/no scale; item scores are summed (range 0–14). Because the ACES-R was added to the battery after the study had begun, data are missing for 168 participants.

#### Motivation

Because some participants were and some were not taking ART at the time of enrollment, we assessed 1) motivation to start taking ART *or* to increase how often participants took ART and 2) motivation to attend HIV medical care on the recommended schedule using a scale created by Rollnick [48], where motivation is conceptualized as how important a behavior or outcome is to an individual and how confident they are they can engage in the behavior or achieve the outcome. Importance of the behavior was rated on a 0–10 scale (e.g., On a scale of 0–10, how important is it to you today to start taking HIV medication, where 0 is not important at all, and 10 is extremely important?), followed by the participant’s confidence that they could engage in the behavior (e.g., On a scale of 0–10, how confident are you that you could take HIV medication- every day, as prescribed- if you started today, where 0 is not at all confident and 10 is extremely confident?). Motivation was coded as the mean of the importance score and confidence scores.

#### Substance use

Substance use patterns were assessed by the World Health Organization Alcohol, Smoking and Substance Involvement Screening Test (WHO ASSIST) [49]. The ASSIST questionnaire is designed to identify people who are using substances in a hazardous way that may be creating harms. We assessed the following domains cross 10 substances (tobacco products, alcohol, cannabis, cocaine, amphetamine-type stimulants, sedatives and sleeping pills, hallucinogens, inhalants, opioids, and ‘other’ drugs): lifetime use, frequency of recent use (past 3 months), and the frequency of experiencing indicators of hazardous use; namely, a strong desire or urge to use; health, social, legal or financial problems related to substance use; interference with role responsibilities related to use; whether anyone else has ever expressed concern about the participant’s use of each substance; and whether the participant has ever tried to cut down or stop the use of a substance and failed in that attempt. The WHO ASSIST provides algorithms for a risk score for each substance: lower risk (occasional or non-problematic use), moderate risk (more regular use), or high risk (frequent hazardous or problematic use). We created a risk score capturing moderate-to-high-risk use (yes/no) for alcohol, cannabis, and other drugs combined (cocaine, methamphetamine, opioid, etc.). IDU history was assessed and coded as a three-level variable: IDU lifetime, but not in the past 3 months (yes/no), IDU in the past 3 months (yes/no), and never injected drugs. We also assessed engagement in any substance use treatment in the past (e.g., outpatient drug treatment, methadone maintenance treatment program, 12 step or self-help meetings like AA or NA), an indicator of past substance use problems (yes/no).

#### Social support and networks

We assessed social support with a 13-item version of the Medical Outcomes Study (MOS) Social Support Survey [50]. The MOS measure assesses five types of social support: emotional, informational, tangible, positive social interaction, and affectionate support. Items were assessed on a five-point Likert-type scale (ranging from 1/ none of the time to 5/all of the time). Scores were summed (range 13–65) and Cronbach’s α = 0.95. Participants were also asked the number of people known to them by name or face who were living with HIV [51].

#### Stigma

ART medication stigma and stigma associated with HIV care were each assessed using a three-item scale adapted from the Patient Medication Adherence Questionnaire [52]. Items (e.g., Taking my medicines reminds me that I have HIV) were scored on a three-level Likert-type scale (disagree, not sure, agree). Scores were summed and ranged from 3 to 9. Cronbach’s α was acceptable for the medication stigma (0.66) and care stigma (0.60).

#### Barriers to HIV care and ART

Subjective, mainly structural, barriers to HIV care and ART, such as lack of transportation, lack of childcare, and distrust of providers, were assessed using a 10-item scale where participants answered yes/no for each [53]. The number of barriers was summed (range 0–10).

#### Outcome variables

Those diagnosed with HIV over a longer period have more opportunity to both stop and start ART and sustain ART and the national guidelines about when to initiate ART have changed over time [54]. To control for variation in the number of years since participants first initiated ART, we included the years since first ART initiation as an offset term.

### Qualitative interview guide

The in-depth interviews followed a semi-structured interview guide collectively developed by the research team and grounded in the integrated theoretical model and the transformative approach, as described above. The interview guide included a series of questions and prompts to uncover and explore past and recent experiences living with HIV and perspectives on engagement in HIV care and ART adherence. The guide had two sections: 1) general explorations of living with HIV and HIV management and 2) an exploration of specific aspects of the larger study. The present study focuses on responses to the former section. Questions focused on structural-level factors, such as stable housing and access to care and ancillary services. Social-level factors included relationships with health care settings and providers (e.g., To what extent have you felt welcome and wanted at your HIV health care setting when you have been taking ART? What about when you have not been taking ART?). Individual-level domains included the participant’s experiences with ART (e.g., Have you ever taken HIV medications in the past? [IF YES] When did you start? How long did you take ART? Can you tell me what else was going on in your life at the time?), perspectives on ART (e.g., Has anything changed about the way you think about HIV medication? [If not on ART], Do you think that there is anything that would make you want to consider taking HIV medications on a regular basis? and emotions about ART (e.g., Had you ever talked about your feelings about HIV medications to your provider before?). Throughout the interview process, the interview guide was updated to reflect newly emergent concepts (e.g., feeling pressured to take ART and its effects). The qualitative interview guide is provided as a supplementary file.

### Approach to the analyses

Consistent with the convergent parallel design, analyses of quantitative and qualitative data were carried out concurrently and independently. Then, we compared, contrasted, and integrated the results from the two analyses [21].

#### Quantitative data analyses

We used descriptive statistics to summarize the outcomes, as well as socio-demographic and background characteristics (age, sex assigned at birth, gender identity, sexual minority status, race/ethnicity, education status, ever incarcerated, number of times incarcerated), HIV-related indices (years since first HIV diagnosis, where diagnosed, number of co-occurring physical/mental health conditions, medical insurance status), and individual- (adverse childhood experiences, motivation to take/adhere to ART, motivation to engage in HIV care, use of alcohol, cannabis, or other drugs at a moderate-to-high-risk level, lifetime and recent IDU, past substance use treatment), social- (social support, number of people known with HIV, and HIV medication and HIV care stigma), and structural-level barriers to ART (ever homeless, perceived barriers to HIV care). Reasons for stopping ART were described using descriptive statistics but not included in bivariate analyses. To estimate associations between socio-demographic and background factors, HIV-related factors, and individual-, social-, and structural-level factors and the two outcomes, we used negative binomial regression models [55] with years since first started ART as an offset. Negative binomial regression is appropriate for count or rate outcomes and allows for the estimation of underdispersion or overdispersion. Estimation of dispersion avoids reliance on an assumption that the mean and variance of the count outcome are equal. Coefficients estimated by negative binomial regression models are log rate ratios, and exponentiating the coefficients leads to incidence rate ratios (IRRs), which describe how a one-unit change in the explanatory variable multiplies the outcome variable rate. The offset term adjusts for individual differences in time at risk for the outcomes. In separate bivariate analysis, the count of ART starts and longest duration of sustained ART (in months) were regressed on socio-demographics, background factors, HIV-related health factors, and individual-, social-, and structural-level barriers to/facilitators of ART. Associations were reported as IRRs. The MASS package [56] of the R statistical computing program [57] was used for the negative binomial regression analysis, including tests of significance and confidence intervals (CIs). All tests of statistical significance were two-tailed, and *p* < .05 was considered significant.

#### Qualitative data analysis

The strategy used a directed content analysis approach that was both inductive and theory-driven [58]. First, a primary researcher trained in medical anthropology analyzed interview transcripts and developed an initial start-code list and operational definitions for each code, informed by the theoretical perspectives guiding the study [10, 13, 15], including codes related to culture and race/ethnicity (e.g., experiences of discrimination, medical distrust) [59]. Then, the primary analyst coded transcripts using the start-code list. Next, two additional trained qualitative researchers coded a subset of the interview transcripts and met frequently with the primary data analyst. Codes were further refined and elaborated upon, and discrepancies were resolved by consensus. After resolution of discrepancies, each transcript was then recoded using the final coding frame. Then, codes were combined into larger themes and sub-themes in an iterative process and in collaboration with an interpretive community made up of members of the research team [50]. Regarding positionality and methodological rigor, the research team was made up of men and women from White, African American/Black, Asian, and Latino/a backgrounds. The primary data analyst was a member of the research team trained as a medical anthropologist and experienced with HIV research, including with this subpopulation of PLWH. Positionality challenges related to sex, gender, race/ethnicity, power, health, SES, and privilege were intentionally addressed throughout the data collection process through reflection and training, which focused on how these types of issues might impact the interviewing process and data analysis [60, 61]. Although we used the random sampling method for the qualitative study component based on the demands of the larger study, we attended to issues of maximum variation in sample characteristics [62] and saturation on primary themes [63] in the data analysis process as one aspect of trustworthiness [64]. Methodological rigor of the analysis was further maintained through an audit trail of process and analytic memos and periodic debriefing with the larger research team, which included PLWH and experts in long-term HIV survivorship and ART adherence, as well as member checking with PLWH; feedback from the member checking was incorporated back into the results [65].

#### Data integration

First, the interpretive community assessed concordance between the quantitative results and the primary themes in the qualitative data. To do so, we used an informational matrix [21, 66] to compare the quantitative and qualitative results at a granular level (variable by variable) and examine areas of convergence and divergence. The primary results were summarized and presented in a joint display table (Table 5).

## Results

### Quantitative results

Table 1 shows participant socio-demographic and background factors. Participants ranged in age from 19 to 65 years (mean = 47 years; SD = 11 years). Most were men (70.1%). Half (50.4%) of male and 20.9% of female participants were sexual minorities. Most were African American/Black (68.6%). More than two-thirds had a high school diploma or higher (70.1%), and approximately half had been incarcerated in the past (58.1%). They had been diagnosed with HIV for an average of 18 years (SD = 9 years). Almost all (98.4%) had taken ART in the past and the majority had health/medical insurance (97.8%).

**Table 1:** Sociodemographic and background characteristics and HIV-related health factors (*N* = 512)

	Mean (SD) or %
Age in years (M, SD)	47.0 (10.7)
Age range [min, max], in years	19.0, 65.0
*Sex, sexual orientation, and gender identity*	
Male sex assigned at birth	70.1
If male sex, cisgender and heterosexual	40.1
If male sex, cisgender and sexual minority	50.4
If male, transgender	9.5
Female sex assigned at birth	29.9
If female sex, cisgender and heterosexual	74.5
If female sex, cisgender and sexual minority	20.9
If female, transgender	4.6
African American/Black, non-Latino/Hispanic	68.6
Latino/Hispanic	27.3
High school graduate/GED or higher	70.1
Ever been in a detention center, prison or jail > 24 h	58.1
If yes, number of times in a detention center, prison or jail	11.3 (16.0)
*HIV History and Physical Health Status Indicators*	
Years living with HIV (M, SD)	18.2 (8.6)
Range of years living with HIV (min, max)	0.00, 30.0
Perinatally infected with HIV	2.3
Diagnosed with HIV in jail/prison	18.4
Diagnosed with HIV in a hospital emergency department	5.5
Diagnosed with HIV in a hospital, as an inpatient	15.6
Has taken ART in the past	98.4
Years since first initiated ART	14.0 (8.5)
Range of years since initiated ART [min, max]	1.0, 30.0
If not on ART at enrollment, number of months since last ART dose	5.0 (21.3)
Range – number of months since last ART dose [min, max]	1.0, 292
Number of co-occurring physical and mental health co-conditions ever diagnosed with	3.9 (2.7)
Covered by insurance or health plan	97.8

Table 2 shows reasons for stopping ART among those who had taken ART in the past. The most common reasons were side effects (54.6%), switching ART regimens (45.7%), having high CD4 and/or low HIV viral load; that is, favorable HIV health indicators (39.3%), and being on too many medications (36.9%).

**Table 2:** Reasons for stopping ART, if ever took ART (*N* = 504)

	%
Did not want medication side effects	54.6
Switched to other HIV medications	45.7
Decided to stop because CD4 and viral load numbers have been good	39.3
Were on too many medications	36.9
Changed doctors or clinics	29.8
The HIV medication(s) initially worked, but then stopped working	25.5
Disclosure concern (did not want anyone to find out that about HIV positive status)	24.2
Prefer alternative treatments	19.0
The HIV medications(s) did not work	18.5
Doctor told you to stop	9.9
Could not afford the HIV medication(s)	7.2
Believed you have been cured of HIV	7.1
Partner or friends suggested you stop taking HIV medication(s)	7.0

Table 3 shows individual-, social-, and structural level barriers to ART. Participants reported an average of 4.4 adverse childhood experiences (SD = 4.2 experiences). Motivation to either start taking ART (if the participant was taking ART at all) or significantly increase how often he/she was taking ART (if the participant was taking ART) was moderate (Mean score = 6.0, SD = 3.3, on a 0–10 scale). Motivation to attend HIV medical care appointments on the recommended schedule was also moderate (Mean score = 6.9, SD = 3.1 on a 0–10 scale). Use of alcohol (45.5%), cannabis (62.7%), and other drugs (69.9%) at moderate-to-high-risk levels were common. More than two-thirds of all participants had been in substance use treatment at some point in the past (72.3%). A modest proportion had injected drugs in their lifetimes but not in the past 3 months (12.7%) and 8.8% had injected drugs in the past 3 months. Most participants had experienced homelessness (89.5%). Participants had started ART a median 5 times in the past. The median longest period of sustained ART was 18 months.

**Table 3:** Individual-, social-, and structural-level barriers to ART (*N* = 512)

	Mean (SD) or %
*Individual-level factors*	
ACES-R score (range 0–14)	4.4 (4.2)
Motivation to start taking ART or significantly increase how often you take ART (range 0–10)	6.0 (3.3)
Motivation to attend HIV medical care appointments on recommended schedule, (range 0–10)	6. 9 (3.1)
*Substance use*	
Alcohol use at a moderate-to-high-risk level	45.5
Cannabis use at a moderate-to-high-risk level	62.7
Use of other drugs at a moderate-to-high-risk level	69.9
Never injected drugs	78.5
Injection drug use lifetime, but not in the past 3 months	12.7
Injection drug use in the past 3 months	8.8
Participated in substance use treatment in the past	72.3
*Social-level factors*	
Current social support (range 13–65)	35.9 (14.8)
Number of people known living with HIV, Median [Q1, Q3]	20.0 [10.0, 75.0]
HIV medication stigma (range 3–9)	5.4 (2.2)
HIV care stigma (range 3–9)	4.9 (2.1)
*Structural-level factors*	
Ever homeless	89.5
Perceived structural barriers to HIV care (range 0–10)	1.4 (2.2)
***Outcome variables***	
Times started ART in the past, range 0–300 times, Median [Q1, Q3]	5 [3, 10]
Longest duration of sustained ART, in months, range 0–297 months, Median [Q1, Q3]	18 [6, 36]

Table 4 shows associations between patient demographic factors, background factors, and individual-, social-, and structural-level barriers to ART and both ART starts and longest period of sustained ART. Factors associated with a higher rate of ART starts, considered a less favorable outcome, were male sex (IRR = 1.37, 95% CI = 1.05–1.77), transgender gender identity (IRR = 2.03, 95% CI = 1.33–3.20), cannabis use at a moderate-to-high-risk level (IRR = 1.27, 95% CI = 0.99–1.63), stigma associated with ART (IRR = 1.08, 95% CI = 1.03–1.15), and stigma associated with HIV care (IRR = 1.07, 95% CI = 1.01–1.14). Factors associated with a lower rate of ART starts were older age (IRR = 0.97, 95% CI = 0.96–0.98), more years since diagnosis (IRR = 0.96, 95% CI = 0.95–0.98), motivation for HIV care (IRR = 0.92, 95% CI = 0.89–0.96), and lifetime but not recent IDU (IRR = 0.58, 95% CI = 0.41–0.84). Factors associated with a longer duration of sustained ART, the more favorable outcome, were Latino ethnicity (IRR = 1.39, 95% CI = 1.11–1.76), motivation for ART (IRR = 1.07, 95% CI = 1.03–1.10), motivation for HIV care (IRR = 1.03, 95% CI = 1.00–1.07), and IDU in the past 3 months (IRR = 1.46, 95% CI = 1.03–2.12). Factors associated with a shorter duration of sustained ART were African American/Black race (IRR = 0.74, 95% CI = 0.59–0.92), alcohol use at a moderate-to-high-risk level (IRR = 0.78, 95% CI = 0.64–0.96), and higher social support (IRR = 0.99, 95% CI = 0.98–1.00).

**Table 4:** Bivariate Associations with ART Starts and Longest Duration of ART from Negative Binomial Regression

	ART Starts			Longest Duration of Sustained ART		
	Incidence Rate Ratio (95% CI)		*p*	Incidence Rate Ratio (95% CI)		*p*
**Age**	0.97	(0.96–0.98)	<.0001	0.99	(0.98–1.00)	0.1828
**Male**	1.37	(1.05–1.77)	0.0143	1.20	(0.95–1.50)	0.1112
Cisgender Sexual Minority vs. Cisgender Het	1.24	(0.97–1.59)	0.0764	1.17	(0.95–1.46)	0.1392
**Transgender vs. Cisgender gender identity **	2.03	(1.33–3.20)	0.0010	0.99	(0.69–1.48)	0.9660
**Non-Latino/Hispanic African American/Black**	1.12	(0.86–1.44)	0.3755	0.74	(0.59–0.92)	0.0072
**Latino/Hispanic**	0.90	(0.69–1.18)	0.4382	1.39	(1.11–1.76)	0.0042
No HS Diploma/GED	0.92	(0.71–1.20)	0.5206	0.88	(0.70–1.10)	0.2418
Ever Homeless	1.46	(0.97–2.14)	0.0536	0.94	(0.66–1.31)	0.7307
Ever Incarcerated	0.80	(0.63–1.02)	0.0654	1.04	(0.84–1.29)	0.6887
**Years Since Diagnosis**	0.96	(0.95–0.98)	<.0001	0.99	(0.98–1.00)	0.0697
Diagnosed in Jail	0.81	(0.59–1.12)	0.1636	1.01	(0.78–1.33)	0.9379
Diagnosed in Emergency Department	0.98	(0.59–1.69)	0.9227	0.85	(0.56–1.35)	0.4629
Diagnosed in Inpatient Care	0.90	(0.66–1.25)	0.5234	1.15	(0.87–1.53)	0.3241
Co-occurring conditions	1.00	(0.96–1.05)	0.9913	0.99	(0.95–1.03)	0.5119
Adverse Childhood Experiences	1.03	(1.00–1.07)	0.0522	0.99	(0.97–1.02)	0.7092
**ART Motivation**	0.97	(0.94–1.00)	0.0835	1.07	(1.03–1.10)	0.0001
**HIV Care Motivation**	0.92	(0.89–0.96)	<.0001	1.03	(1.00–1.07)	0.0480
**Alcohol at Moderate-to-High-Risk Levels**	1.09	(0.86–1.38)	0.4791	0.78	(0.64–0.96)	0.0173
**Cannabis at Moderate-to-High-Risk Levels**	1.27	(0.99–1.63)	0.0453	0.90	(0.73–1.11)	0.3196
Other Drugs at Moderate-High-Risk Levels	1.04	(0.79–1.34)	0.7832	0.94	(0.75–1.18)	0.6128
**Lifetime IDU, but not in the past 3 months**	0.58	(0.41–0.84)	0.0026	0.98	(0.72–1.35)	0.8808
**IDU in the past 3 months**	0.81	(0.55–1.25)	0.3214	1.46	(1.03–2.12)	0.0382
Lifetime Substance Use Treatment	0.86	(0.66–1.13)	0.2689	0.90	(0.71–1.13)	0.3560
**Social Support**	0.99	(0.99–1.00)	0.1549	0.99	(0.98–1.00)	0.0020
PLWH Network Size (natural log)	1.00	(1.00–1.00)	0.0937	1.00	(1.00–1.00)	0.2111
**ART Stigma**	1.08	(1.03–1.15)	0.0036	0.97	(0.93–1.02)	0.2780
**Care Stigma**	1.07	(1.01–1.14)	0.0127	0.95	(0.91–1.01)	0.0630
Perceived Barriers to Care	0.96	(0.91–1.02)	0.1614	0.96	(0.92–1.01)	0.1271

### Qualitative results

#### Overview

Most participants had long histories of living with HIV, and their narratives demonstrated that ART was generally acknowledged as one’s best chance for a long and healthy life, even if perspectives on ART reflected ambivalence. Yet, stopping ART was common, for both short intervals and longer stretches of time. Participants identified a set of related or interdependent factors that played a role in their stopping ART, restarting ART, and/or sustaining ART in times of hardship when the risk for discontinuation may be high. In fact, throughout their interviews, participants described that serious barriers to practicing self-care and managing health were routine, including barriers to engagement along the HIV care continuum. At the same time, factors that facilitated or supported the management of health and wellbeing were less common. The most common themes present throughout the interviews were related to the following domains: substance use, housing and other material factors, social support, quality healthcare and social services, and mental health. While the importance of these factors in the lives of African American/Black and Latino PLWH is well-documented, the present study’s results illuminated the mechanisms by which these barriers and facilitators operated, from the perspectives of the population under study, and how these barriers and facilitators were experienced. Importantly, participants described how these barriers and risk factors were continuously shaped by institutional racism, HIV-related and other types of stigma, and other forms of social inequity. We expand upon each of these interrelated themes below. We focused results mainly on barriers to and facilitators of ART stops/starts and sustained ART, and factors that related to engagement in HIV primary care are embedded within this primary focus. In reporting the qualitative results, we used pseudonyms and also changed or obscured participants’ identifying details such as age and/or years since HIV diagnosis to maintain their confidentiality.

#### Substance use

Relationships among participants’ substance use patterns, engagement in HIV care, and ART uptake and sustained ART use were a complex, multifaceted, and often idiosyncratic phenomenon, consistent with the integrated theoretical model that highlights multi-level influences on behavior. Nonetheless, almost invariably, participants cited issues related to episodic and sometimes heavy or hazardous substance use among the primary obstacles to engaging in HIV care, starting ART, and/or sustaining high levels of ART adherence. During periods of active substance use, erratic sleeping patterns, extended periods of time away from home, and efforts to procure substances made it difficult to take medications on a regular basis, often leading to an avoidance of HIV care and cessation of HIV medications altogether. Commonly, the need to maintain consistent substance use necessitated selling or “diverting” HIV medications [67], which in many areas had a street value of up to several hundred dollars per bottle. In fact, in the setting where the present study was based, pharmacies routinely solicited the sale of ART from PLWH from low-SES backgrounds, even though this practice is illegal [68, 69], and it was often difficult for participants with material needs to decline such offers. This inducement to sell ART was experienced by many participants as a type of structural barrier to sustaining ART. Garrett was a Black man in his late 40s who was diagnosed with HIV as a teenager. He described how his use of substances challenged his ability to manage ART, which, in turn, resulted in a discernable decline in his physical wellbeing:

I was getting high and I was selling my meds. I was selling my meds and yes. And I was just getting sicker and sicker, and you know. I lost a lot of weight. I knew my body […] didn’t feel right. I was having aches and pains, and I knew it was because I wasn’t taking my meds. But you know, I was caught in the grips. And I was still getting high, and I was still selling the meds. And I was just getting sicker and sicker.

It was common for participants to report that substance use was intricately related to other material, emotional, and relational challenges. Primary among these were unstable housing, lack of employment, unstable or challenging romantic and familial relationships, and incarceration. Among these, HIV and other related stigmas, including stigma associated with substance use, were the most common social-level barriers participants cited as causes of difficulty sustaining ART use. One common mechanism by which stigma operated was by creating social isolation, which, in turn, contributed to ART cessation. Ernie, a Black man in his early 40s who had been living with HIV approximately a decade, drew a direct line between substance use and, his quote implies, substance use-related stigma, social isolation, and ART discontinuation:

Everything was just haywire. Relationships played a big part in me not taking medication. Yeah, the pressure. My family. Me battling my kids, battling with drug addiction, alcoholism, trying to find who I am at a late age. Oh my God, stuff just happened, stuff I hadn't experienced before. So, my mind started to get mushy, and I started to just go and isolate myself from everybody, you know?

In addition to the everyday challenges that substance use presented to sustaining ART, it was common for participants to express the need to stop or “take a break” from ART due to concerns that HIV medication might interact with substance use to cause long-term adverse health effects. This included fears of organ damage, which many were concerned was already in motion as a result of chronic or intermittent heavy substance use. Harold, a Black man in his early 50s who had been living with HIV for approximately a decade, noted:

I’m too busy doing other things because I’m not clean and sober and I, you know, can’t take it [ART]. […] I know that I need to take it. But, because I’m indulging in what I indulge in, I’m not going to take it because it’ll do more damage than good … alcohol and the pill … Yeah [substances and HIV medication] don’t work [together].

Substance use was often closely associated with individual and intergenerational trauma, chronic stress, and instability, and served as a means of managing negative emotions and experiences, which in themselves also frequently made sustaining HIV medication exceedingly difficult. Yet, discontinuing ART also tended to cause negative emotions among participants. Thus, periods of active substance use were associated with fears of long-term adverse side effects, and periods of ART discontinuation were characterized by fears of the effects of untreated HIV.

Conversely, starting and/or sustaining ART were closely associated with a reduction or cessation of substance use or the development of successful harm reduction strategies. Restarting or sustaining ART typically involved a number of steps, the first among them developing and/or reestablishing supportive social relationships as well as maintaining emotional and material stability, which allowed participants to keep a consistent schedule. For those participants who did not wish to (or who were unable to) abstain completely from substance use, the understanding that ART was still effective when one was using substances [70, 71] was critical to the decision to restart HIV medication. Benito was a Latino man in his mid-50s who was diagnosed with HIV 20 years prior. For Benito, adherence to HIV medications was made especially difficult by his need to sell his ART to support his substance use needs, but also because he labored under the assumption that substance use either lessened or cancelled out the effects of HIV medication. Upon learning otherwise, however, Benito noted the following:

I still use drugs. […] I know that I really do need to stay focused on taking that medication regardless of whether I want to stop [using ART] or not. Breaks aren't good. I used to do that. I would take a break for about four or five months, sell the medication, use it for drugs, break myself down a little bit, then stop, [re-start ART], and go and do it again. And now I don't want to do it anymore. That's burning me out.

Rodney was a Black man in his late 40s who had been living with HIV for approximately a decade and who described himself as struggling with alcohol ever since he was first diagnosed. He described his own personal strategy for sustaining ART use while also using substances as follows:

Like I said, even though I heard that you don’t mix [HIV] medications and alcohol or drugs together, what I was told was even if you are going to get high or whatever, still do them [ART], you know what I’m saying? Still take your meds, you know what I’m saying, because it’s important, but I look at it like this. If I know I’m going to go drink, I’ll try to take them before I go do my drinking. […] Like an hour or two. […] But regardless of how I feel or what I’m doing, the medication is going to keep me alive. Which also I know that they may not work as effectively as they’re supposed compared to you not using [substances], you know what I’m saying? But at least you still have some in you; some of the medications. […] But I learned even if you are going to get high or drink or what have you, still take your medications; that’s what I learned.

After eventually feeling comfortable enough to speak with health care providers and peers about his desire to take ART and also to use substances, Rodney, like many participants, decided to restart ART and found it possible to sustain ART with high levels of adherence, even when using alcohol and/or drugs. Yet, many participants, including Harold, described above, assumed ART and substances were mutually exclusive. Moreover, it was challenging for participants to discuss substance use with health care providers, and stigma related to substance use was a potent barrier to ART. Franklin, a Black man in his early 40s who had been living with HIV for a little over a decade, noted the following:

I never went there [to the HIV care setting] high, but the fact [is] that it matters that knowing that she [the health care provider] knew that I was doing coke, you know? She looked at me in a different way. You faced a lot of that – oh of course. I was like, okay, well, since I can’t come to the treatment I’m not going to [take] the medication. […] It discouraged me. I just stopped taking medications completely.

Over time, emotional and perhaps physical fatigue set in for many participants with heavy substance use. As Lawrence, a Black man in his mid-50s who was diagnosed with HIV two decades ago, noted, “Most people who use drugs and all that stuff, they’re basically tired because the drug damage really did. So, these people are basically tired.” These findings reflect the integrated theoretical model; namely, that substance use-related barriers to ART operate simultaneously at multiple levels of influence, and that among these, structural- and social-level factors played an important role both in contributing to substance use patterns, and also to how substance use acted as a barrier to ART.

### Housing and other material factors

Material and financial insecurity, particularly the lack of adequate and affordable housing, presented participants with another set of structural-level barriers to sustaining ART. For instance, Clarence was a Black man approaching the age of 50 who was diagnosed with HIV nearly 20 years ago. He described how his current living conditions in a single room occupancy residence for PLWH contributed to a sense of instability, which led directly to fears he would be unable to manage his HIV medication:

I moved to this room, it’s horrible, in the Bronx. It’s very hot, you can’t cook, you have to buy every meal. […] Because I can’t cook, it’s so much more expensive having to buy every meal, every day of the week. They strictly forbid cooking, they do all these inspections, they don’t even want to see a hotplate, a toaster oven, a little tiny microwave. […] The whole kitchen is disconnected, the stove, everything, because it used to be an apartment, but they made it a [two room type of] apartment. So, the living room’s empty, the kitchen is just there, and you share the bathroom with one other person. I want to get out of there as soon as possible. I’m just used to being in an apartment [with a kitchen]. Unfortunately, this [move to the single-room occupancy residence] happened, but [living in] this place it kind of makes it hard, but I’m trying to keep on the up. Because I know if I go down, then my adherence is going to get thrown off.

Thus, Clarence was successful in sustaining ART, even when aspects of the larger environment threatened to weaken his health habits. Clarence’s comments highlight both the salience of autonomy, and the problems that arise when autonomy is restricted, and the drive for competence (albeit thwarted in this case) in this type of housing setting, consistent with the integrated theoretical model. Similarly, when describing the relationship between substandard housing and social isolation, Garrett (introduced above), noted how the only housing he was able to secure was commonly known to be a hub for various illegal activities, which led directly to self-isolation:

[When I was home, I was] doing harm to myself, yes, because when I was home it was just like every time the doorbell rung I would open it because I knew it was drugs or money. You know, so I was always putting myself in harm's way.

As was the case with many other participants, Clarence and Garret were each keenly aware of how the sub-optimal quality of their living situations put them at high risk for being unable to sustain HIV medication use. Yet, their narratives also highlighted or suggested the ways they coped with these contextual challenges, including by being aware of how the housing environment could undermine their personal health goals.

In the sections above we described how participants commonly diverted ART to fund substance use needs. Participants also commonly reported the need to sell HIV medications to pay for basic life necessities, such as food, clothing, bills, and other daily living expenses. Mona, a Latina woman in her 30s who had been living with HIV for a decade, shared the following experiences:

I try to take my meds. I'm not going to lie, sometimes I sell them because I need the money. But my health is very poor right now. Well, my CD4, last time it was 28 [a very low level], and that was in April. I know I don't want to die, it just sometimes when you need money for the kids, I have to think of something. It's not like he's [her romantic partner] going to go get the money. […] Honestly, I just give it [ART] to my friend, she gets $60 for each pill. So, when you're broke $60 is okay, but I need to take my meds. Because I want to be around, you know, I got kids. But when you ain't got nothing to eat, you know, you got to make a sacrifice and then he [her romantic partner] gets mad.

Thus, consistent with the multi-level integrated theoretical model, material precarity unrelated to substance use can drive ART diversion, even when a participant’s motivation to take ART was high. Raul was a Latino man in his late 50s who had been living with HIV for over 20 years. He described self-isolation, which he connected to socio-economic barriers and institutional racism that he viewed as a direct response to his ongoing involvement in the criminal justice system, unemployment (which, of course, greatly reduced his material resources), and a desire to avoid disclosing these problems to his family. As a result, Raul found it all but impossible to regularly seek HIV care or to take his HIV medications:

I got depressed. […] I was starting running through the streets. And I relapsed. Parole was chasing me down. I had a good job. I used to work [in the food industry]. And parole told me, oh, you're making too much money. We feel that it's not appropriate. I'm like, I completed a drug program. Now I find a good job. Now you're telling me I'm making too much money [and] to quit. I'm not going to quit. So, we got into a debate. […] And they kept harassing me. So, I told them to go F-you and do what you got to do when you catch me. So, I figure if I stay in the room, it's going to make it easy for them to get me. So, I decided to run to the streets when I could have ran home to the family. But I didn't want them to know that I was having problems with parole, about the job; my kids were going to the job, they were happy. Everybody was happy for me, you know. Wow, you got a good job, you're doing great. But I kept a lot of stuff to myself.

Indeed, the deleterious effects of material barriers such as unstable housing, unemployment, and the inability to meet basic needs commonly put sustaining HIV medication and/or attending regular visits to a healthcare provider out of reach. Conversely, participants who had obtained stable and high-quality housing credited this achievement as triggering a cascade of positive effects in other parts of their lives, including enhanced self-esteem and strong social connections, thereby enabling them to reinitiate and/or sustain ART with high levels of adherence. Again, these findings highlight how barriers to ART interact across multiple levels of influence, consistent with the integrated theoretical model.

### Social support

Being diagnosed with HIV was frequently experienced as a “gut punch,” often accompanied by a sense that nothing in life would ever be the same. Upon learning of their HIV diagnosis, many participants reported experiencing a profound sense of anxiety regarding how their HIV status might affect familial, romantic, and other social relationships. For many, this led to prolonged periods of social isolation, depression, and a sense that ART was simply not worth the effort. Indeed, the lack of adequate social support or, in the language of self-determination theory, relatedness, a critical social-level barrier, often left PLWH facing numerous other hardships. This included feeling as if their experiences were uniquely disadvantaged and stigmatized, thereby exacerbating existing feelings of dejection and hopelessness. Corea, a Latina woman in her early 60s who had been living with HIV for approximately two decades, described how bouts of extreme loneliness influenced her desire to stop taking HIV medications:

At the beginning, I used to cry because it was hard for me being depressed and not taking the medications and not having the support when I get home. […] I do have it always in my mind that I'm going to take it [ART]. It's important. But maybe it has to do with my moods. I live by myself and it's hard when you don't have no one home to talk to. I have children and I usually call them but they're always busy. I call them before they start to work or after, around 8 p.m. It's hard to live by yourself.

Some participants had previously faced relatively few barriers to ART adherence. Commonly, the sudden loss of one or more close relatives, friends, or romantic partners almost immediately precipitated ART discontinuation. Jocelyn, a Black woman in her late 20s who was perinatally infected with HIV, recalled the reason she stopped taking ART with precision:

When I grew up, I was taking my medicines on time, all that, until my dad passed away and my brother. That's when I stopped taking my medicine regularly, and that's when everything just started pulling apart. And now it's just like, what else?

In these and other instances, participants described in detail how their willingness and/or ability to manage HIV medications were often tied directly to the degree to which they felt socially connected with others – findings that align with the domain of relatedness in the integrated theoretical model.

Participants often found ways to develop meaningful connections with other groups or individuals who shared similar experiences, both related to HIV and otherwise, and the resulting support played a significant role in their decisions and abilities to start and/or sustain ART, again, consistent with the construct of relatedness. For Marion, Black man in his mid-40s who was diagnosed with HIV approximately a decade ago, the peer support he received in a social service agency encouraged him to stop selling his HIV medications. This, in turn, resulted in his achieving an undetectable HIV viral load level, the ultimate goal of ART:

Because I learn from other people's life stories. You know what I mean? Because sometimes you be talking, you know, like what you go through. You be thinking it's just you going through it. […] There would be a lot of people [there] that simply would be worse. And those are things that helps me to move forward, because I know – I'm a guy. I face reality.

For other participants, the impetus to restart and eventually sustain ART with high levels of adherence came about as a direct result of (re)establishing connections with friends, family, and loved ones, who, in turn, served as a reason to live. Derek was a Black man in his early 50s who had been living with HIV for over two decades and who had experienced street homelessness up until a short time before being interviewed. He noted that reconnecting with family led to a period of critical self-reflection, wherein he ultimately made the decision to begin taking HIV medication again:

I was – like I was just telling you, self-destruct, selling my meds, smoking crack, smoking so much crack that I was about ready to die. I didn’t care. And through coming through [a social service setting] and listening to myself speak and telling [program staff] everything that’s been going on, and then how I’ve been doing with my medication and all this other mess, was making me look at – damn, do you want to die? Damn. You up here now with your niece and her kids. Don’t you want to see them graduate from school, high school, going on to college? I was like, yeah, I do. I really do. I’d like to see them grow up and get married. I’m only [in my early 50s]. I still got some time left here on this earth. As long as I take my medication. You know. As long as I take my medication, I’m good. I’m doing great with that right now. I’m doing great. […] Now I take it. I don’t sell it. Why should I sell it? I’m not trying to leave my nieces and my nephews. So that’s where that’s at.

Derek’s statement echoed that of many other participants throughout this study in locating an important source of motivation for health in community interaction. It also highlighted that participants typically viewed ART as their best chance for a long and healthy life and greatly feared or dreaded dying from HIV-related causes. At the same time, the will to live could be weakened by year after year of adverse circumstances including poverty, poor quality housing, stigma, and substance use. Indeed, the ability to cope with and/or overcome social isolation played a critical role in the willingness and ability of participants to successfully manage HIV and other adverse health conditions. As Alysse, a Black woman in her early 40s who was diagnosed with HIV approximately 15 years prior, noted: “My son is my reminder. You know, and when I pop my pill, I always say this is my healing. And it is. Because I’m undetectable.”

### Quality healthcare and social services

For many participants, medical distrust played a critical role in the decision whether to restart ART or not and influenced whether they were willing to engage in HIV care at all. The majority of participants in this study detailed years of substandard health care experiences, which they attributed directly to sociodemographic characteristics such as race/ethnicity, sexual orientation, social class, and one’s past or present substance use history. In some cases, participants experienced discrimination as a result of these characteristics. Jared was a Black man in his early 60s who was diagnosed with HIV approximately 20 years before. He expressed frustration with the healthcare system and viewed HIV clinics and pharmacies as unconcerned with his health to the point of viewing him like a “laboratory rat”:

I’m just a little fed up, that’s all. You know? And you know, I’ll keep it real with you. I don’t feel like going into any of these buildings because they’re trying to kill me. But there’s going to be no end to that. […] So I’m going to start making some moves, try to get to another clinic. I have to, because if I stay [at my current clinic], I see myself in some real trouble.

Other participants, however, discussed how finding high-quality healthcare dramatically increased their willingness to engage in HIV care and re-start HIV medications. For many, this meant simply finding a healthcare provider with whom they felt valued and respected. Alysse (introduced above) had suffered from post-traumatic stress disorder for years, leading her to avoid HIV care for long periods. Yet, at one point, Alysse located an HIV clinic where she did not experience judgment from health care providers, despite having an “unacceptably” HIV high viral load level, which she assumed would be seen as indicative of her “poor health habits.” Alysse recounted the following:

My [new doctor], she paid attention. She sat down with me initially and asked me what my experiences were over the years, which I thought was very good. Because nobody ever starts from the beginning to really understand who you are. So that was a plus. And then just making sure that, you know, I'm up on my care. If I don't go to an appointment, I get a phone call. From my doctor. Not the assistant. Not the secretary. From the doctor, herself. She calls me and that shows me that you care, you actually care. And she knows that I was less than strong at that moment, so she was just trying to give me hope. And things like that help.

Indeed, participants commonly noted that their decisions to seriously consider re-starting or sustaining ART with high levels of adherence were predicated in part on their abilities to speak frankly with a healthcare provider about their own personal situations, to be treated as a valuable individual and a whole person, without racial/ethnic or other forms of discrimination, and to have their autonomy supported in the process, consistent with both critical race theory and self-determination theory, aspects of the integrated theoretical model.

### Mental health and the importance of self-reflection

Participants characterized long-term HIV survivorship as alternating periods of successful HIV management followed by times of struggle and the need to take a break from ART. Further, citing depression and anxiety in particular, the majority of participants noted that years of constantly shifting combinations of substance use, unstable housing, social isolation, substandard and discriminatory healthcare and social service experiences, and various other forms of institutional racism had a profoundly negative effect on mental health, often resulting in emotional exhaustion and a diminished will or capacity to successfully manage their HIV. Like many participants, Alejandro, a Latino man in his early 30s diagnosed with HIV a decade prior, regarded emotional fatigue, the source of which was difficult for him to describe, as one of the primary reasons for not taking his HIV medications:

I know I need to start taking them [HIV medications] faithfully. I don't know, I just didn't feel like - I was eating right and doing everything else I was supposed to be doing but I just didn't feel like taking them. It's a weird thing. It's a weird feeling. It's not for a particular reason. […] You just get tired. I don't know how to explain it. I don't know the reason that I get tired of taking it. I don't have no symptoms or side effects either. I just get tired. Don't have no symptoms or side effects either. I just get tired. It's always weird because my partner has to take his and he still be taking his and picking his up. He's like, "I don't think I've seen you take your medication." I was like, "No, I needed a break. [But] I'm going back on it."

Similarly, Clarence (introduced above) described the profound effect that depression had on his abilities to take ART. He recalled voluntarily puncturing the foil seal on his HIV medications to discourage himself from selling his ART doses to others. Yet, he often could not overcome the emotions and feelings of depression that prevented him from adhering to his medication and, at the same time, did not feel he could do anything to relieve his depression, with the exception of marijuana:

Now the other part is – that I can’t do anything about, is if I get to my depression or I just – because there’s been times the bottle has just been sitting there, and I didn’t sell it, and I still didn’t take it. That’s just the only battle. And that’d be my mental health issues, and hopefully I could stay on top of that. The weed helps. You know what I mean? I’m just being real. You know, that’s the only thing [I can do], that punch in the bottle [to discourage himself from selling his medication]. But that is not going to change [anything about his depression].

Indeed, for many participants, daily strategies designed to help successfully manage their HIV medications proved largely ineffective during periods of acute depression and anxiety.

Despite experiencing these and other difficulties with ART adherence, however, many participants noted that being given the tools and space necessary for addressing mental health issues in social service settings dramatically improved their abilities to manage HIV. Darryl (introduced above) recalled the utility of guided self-reflection and positive feedback, as well as the ability to develop the tools necessary to deal with past and present trauma in a manner that supported his autonomy, competence, and relatedness, consistent with self-determination theory in the integrated theoretical model. This, in turn, led directly to his decision to restart and sustain HIV medication with high levels of adherence:

I had made the decision that I was no longer going to do it [take ART] anymore, but just coming to this program and beginning to reevaluate my life and my choices, I began to say, wow, this [not taking ART] is not doing anything but hurting myself. At the end of the day, I do want to live. I want to be as healthy as possible. And I made the decision back in October to start taking medication again, and ever since I've been on medication every single day. I haven't missed a dose. I'm undetectable. I really don't think that I would ever be able to say that that was part of my life now, but it is. […] It was the counselor. It was actually somebody sitting down with me, just talking with me, and just allowing me to share and really talk about. “What type of goals you have for yourself in life?” “Where do you see yourself in five years?” I've never really sat down and began to think about something simple like that. And that was the beginning. Then I was given the tools to continue on. Then I was connected to a person who's been through this, and I was able to share war stories. They'd tell me yeah, you're not the only one, but it does get better. It was just icing. Each layer was just another icing on top of the cake.

Marion, introduced above, also discussed how receiving ART adherence support services grounded in the motivational interviewing approach [72] and harm reduction [73] afforded him the space to consciously consider his relationship with his HIV medications and to finally understand the ways the tolerability, convenience, and effectiveness of HIV medications have changed over the years:

I told [my counselor], I said I'm coming to the point where I'm going to get back on meds. You know, I just have to make sense in my head that this is going to help me. You know? I've been on AZT, DDI, Crixivan [various ART medications]. I've been on – I used to take one pill, 27 pills, a day, of one medication. That's when they had the different bottles separate. So, you know, it got to the point where, you know, I'm like why am I taking all this shit, you know? And then you would feel the side effects when they erupt in your stomach. You know, now [new ART regimens] made it real simple. One pill a day.

Recalling his experiences with the same social service setting, Darryl (mentioned above), summed up his experiences being encouraged to engage in sustained self-reflection in a similar manner:

Today, those same dilemmas [that were there a year ago] are still there. Like, none of them have changed, but I've definitely changed a lot. You know what I'm saying? In my view of things, how I take care of myself, and how important it is to me to be medicated and stay healthy – and really almost taking my power back, my life, my control. So, it was really a big whirlwind. Even now thinking about this, like wow, honestly and truthfully somebody saved my life for [a] $10 [gift card for referring new clients to the setting]. That's hella nice, you know what I'm saying? But just to think about that, it's just a lot.

Participants noted that nearly all the barriers to engaging in HIV care or adhering to HIV medications the faced were related to mental health issues in some way, which, in turn, both influenced and were influenced by other barriers mentioned above (e.g., substance use, housing, social support, and quality health care), consistent with the multi-level integrated theoretical model. Given the proper context, resources, and supports, however, many noted that these barriers were ultimately surmountable.

### Integration of quantitative and qualitative results

The interpretative community compared quantitative findings to qualitative findings, working domain by domain, and created a joint display table by consensus (Table 5). Because the qualitative in-depth interviews were semi-structured and therefore allowed for exploration of emergent themes, we did not assume results from the qualitative effort would reflect every quantitative domain. We highlight a subset of the integrated findings presented in Table 5 in this section. First, overall, the qualitative findings provided richness and context to quantitative results. For example, regarding sociodemographic characteristics, quantitative results identified several specific characteristics associated with a higher rate of ART starts (e.g., younger age, male sex, and transgender gender identity) and with the maximum duration on ART (Latino ethnicity associated with a longer maximum duration of sustained on ART and African American/Black race with a shorter maximum duration of sustained ART). As shown in Table 5, the qualitative results highlighted how perceived discrimination based on sociodemographic characteristics contributed to medical distrust, which then acted as a barrier to HIV care and ART. In quantitative results, motivation for ART and motivation for HIV care were both associated with more favorable outcomes. Qualitative results resonated with these results: Motivation was a foundational aspect of various aspects of HIV management and was necessary but not sufficient for HIV management, and support of autonomy was associated with motivation and was an important value for this subpopulation of PLWH, but autonomy not always fostered by service settings. Substance use was an important correlate/predictor of the two outcomes. Quantitative results yielded associations between specific substances and outcomes, while qualitative results described aspects of substance use and their effects on HIV management more generally.

**Table 5:** Joint display comparing quantitative and qualitative findings

Domains of main quantitative findings	Summary of quantitative findings	Summary of qualitative findings
Sociodemographic characteristics	– More starts (the worse outcome) associated with younger age, being male, and transgender gender identity – Longer maximum duration on ART (the better outcome) associated with Latino ethnic background – Shorter maximum duration (the worse outcome) associated with Black racial background	– Participants reported inadequate or even discriminatory health care experiences they attributed to race/ethnicity, social class, sexual orientation, and substance use patterns – These experiences contributed to medical distrust, which was a major barrier to HIV care and ART – *Qualitative results did not reveal major differences between African American/Black and Latino PLWH*
Years since diagnosis	– More starts associated with fewer years since diagnosis	– Emotional fatigue associated with long-term HIV survivorship was common – Giving up on HIV management for periods was common, followed by periods of recommitment to HIV care and ART – *Possible discrepancy between qualitative and quantitative findings*
Motivation for ART and HIV care	– More starts associated with lower motivation for HIV care – Longer maximum duration on ART associated with higher motivation for ART and HIV care	– Motivation was a foundational aspect of HIV management and necessary but not sufficient for HIV management – Motivation was either fostered or eroded/overwhelmed by participants’ larger life contexts and life events – Autonomy was associated with motivation and was an important value for PLWH but not always supported by service settings
Substance use including IDU	– More starts associated with higher-risk cannabis use – Fewer starts associated with lifetime (but not recent) IDU – Shorter maximum duration on ART associated with higher-risk alcohol use – Longer maximum duration on ART associated IDU in the past 3 months	– Substance use (SU) was a primary obstacle to HIV management – The understanding that one can take ART while using substances was critical to sustaining ART, but many PLWH do not know or fully understand they can take ART while using substances – In the context of poverty, PLWH commonly sell or “divert” ART to support SU, and corrupt pharmacies initiate these transactions – SU was disruptive to stability and contributes to social isolation – SU was one component of “taking a break from ART,” a strategy PLWH used to manage fear of ART toxicity – Those with past and recent trauma reported substance use as a coping strategy – Reductions in or cessation of SU promoted re-establishment of social ties, emotional and material stability, and a more consistent schedule, which supported HIV management – Harm reduction was found helpful and consistent with the desire for autonomy and support – *Qualitative findings did not address IDU*
Social support	– Longer maximum duration on ART associated with less social support	– Lack of social support was associated with poor HIV management – Loss of a close other commonly precipitated stopping ART – Peer support and peer modeling were critical to restarting ART and effective ART management – Insufficient social support contributed to “self-destructive” behavior, including social isolation, loss of the will to live, and “not caring” about oneself, which eroded motivation and ability to take ART – *Possible discrepancy between qualitative and quantitative findings*
Stigma re: ART, HIV care	– More starts associated with higher medication stigma and HIV care stigma	– Stigma reduced successful HIV management and frequently resulted in social isolation – Stigma impeded disclosure of HIV status to others, which impeded HIV management
*Major themes from qualitative results not found or not examined in quantitative results*		
Homelessness and housing	– Unstable or substandard housing and other forms of material precarity were major contributors to a sense of instability – Unstable or substandard housing contributed to social isolation – Housing that is unstable or substandard is often a hub of drug selling and other illegal activities, which served as a trigger for PLWH wishing to avoid or reduce substance use – In times of hardship and/or when substance use escalates, PLWH commonly sell or “divert” ART to pay bills – Housing status was one aspect of material precarity, but housing was vital for stability, wellbeing, and to support ART use	
Adverse childhood experiences	– Those with past trauma reported substance use as a coping strategy – Sustained self-reflection helped participants deal with trauma	
Aspects of health care settings	– Health care settings were experienced as a means of making money off patients, and this discourages engagement in care – High quality clinical care, medical care, and social services can foster resilience and engagement in HIV care and ART	

Second, qualitative and quantitative findings appeared to be discrepant in some cases. For example, quantitative findings showed that a higher rate of starting ART was associated with fewer years since diagnosis. Yet, qualitative findings highlighted the need to take breaks from ART, including related to emotional fatigue associated with long-term survivorship. Further, we found in quantitative results that a longer maximum duration of sustained ART was associated with less social support – a counter-intuitive result. Qualitative results highlighted the deleterious effects of low levels of social support and of social isolation and uncovered some of the ways insufficient social support affected them and reduced engagement in HIV care and ART, including by contributing to self-destructive behavior and/or loss of the will to live. Lastly, we found several major themes in the qualitative results not reflected in the quantitative results. For example, unstable or substandard housing, including single room occupancy settings, was a major contributor to a sense of instability. Results described how sub-standard housing interferes with HIV care and ART, such as by exposing PLWH to substance use and thereby encouraging PLWH’s substance use or exacerbating substance use problems and by contributing to social isolation. Overall, the two data sources were complementary, and qualitative results added context, depth, and complexity to our understanding of the mechanisms by which the relationships found in quantitative results may operate. (The integrated results are interpreted in the Discussion section below.)

## Discussion

The present study sought to advance the field of long-term HIV survivorship by uncovering and exploring the factors that contribute to African American/Black and Latino PLWH stopping, restarting, and sustaining ART in times of hardship when the risk for discontinuation may be high. Indeed, very little is known about the experience of living with HIV for a decade or more [4], and PLWH poorly engaged in HIV care appear to be under-represented in research compared to their well-engaged peers [42]. Because racial/ethnic disparities in HIV care continuum engagement are both serious and persistent, we focus on African American/Black and Latino PLWH from low SES backgrounds [74, 75]. Study findings are consistent with the core tenets of critical race theory [27], which underscores the importance of experiential knowledge of populations such as African American/Black and Latino PLWH to better understand the links among structural factors such as institutional racism and ART non-persistence. Indeed, participant narratives repeatedly highlight how racialized norms negatively shape their interactions both in and out of healthcare settings, and as a result compound already existing challenges to re-starting and sustaining ART. The mixed-methods approach proved useful to uncover factors that contribute to stopping, starting, and sustaining ART, including potentially modifiable or addressable factors. The present study also advances the field by describing the complex mechanisms by which risk and protective factors operate, in the voices of African American/Black and Latino PLWH who have lived with HIV for almost 20 years, on average, who had first been prescribed ART 14 years ago, on average, and who are not well engaged in HIV care and do not evidence undetectable HIV viral load. The theory of triadic influence proved useful in drawing attention to structural- (e.g., SES, housing) and social-level (e.g., stigma, social support) influences, in addition to the individual-level factors that are often most apparent to PLWH themselves. Findings are further consistent with self-determination theory, including the importance of individual autonomy in HIV self-management, and highlight the critical role of motivation as a necessary, but not sufficient factor in HIV-related health care and ART behavior.

We found that among this subpopulation of PLWH, stopping and starting ART is common, along with, for many, at least one substantial period of sustained ART. We found both stopping/starting and sustaining ART are influenced by aspects of the larger social and structural environments, and substance use is a major barrier for both starting and sustaining ART. Overall, quantitative results provided a clearer delineation than qualitative results between factors associated with starting ART and those associated with sustaining ART, and qualitative results provide richness, depth, and context to these quantitative results. However, contrary to expectations, the specific factors in the quantitative analyses associated with stopping/starting are not always those associated with sustaining ART. Common factors associated with fewer starts and a longer maximum duration of sustained ART, the more favorable outcomes, included motivation for HIV care and/or ART and IDU (lifetime or recent). Common factors associated with more starts and a shorter maximum duration of sustained ART, the less favorable outcomes, were substance-use related (cannabis or alcohol). Race/ethnicity was not associated with the number of ART starts, but Latino ethnicity was associated with a longer maximum duration of sustained ART, and African American/Black race was associated with a shorter maximum duration, consistent with the literature that indicates that African American/Black PLWH experience the greatest barriers to engagement along the HIV care continuum compared to other racial/ethnic groups [3, 4, 53]. Starting ART may involve decisional processes more than sustaining ART does, and can be prevented or delayed by contextual barriers. In particular, stopping/starting ART, but not sustaining ART, is strongly associated with stigma associated with both care and ART. This is consistent with the existing literature on ART initiation, which suggests avoidance of ART is one way to manage HIV stigma and the emotions associated with HIV and ART and to prevent disclosure of HIV status to others [76, 77]. Those who initiate or re-initiate ART may have better adapted to HIV and found ways to manage stigma than their peers. Similarly, those with more years living with HIV and older PLWH have lower rates of stopping and starting ART, suggesting that PLWH build critical psychosocial capabilities and gain access to resources needed to manage ART as years living with HIV accumulate and PLWH mature [78, 79]. Taken together, findings suggest that processes that drive or buffer stopping/starting ART and sustaining ART are similar in some respects but not others. We interpret the findings below, highlighting associations with stopping/starting and sustaining ART separately, or as a unitary phenomenon in other cases, as is appropriate.

Taking quantitative, qualitative, and integrated findings together, we found a number of structural and social factors that bolster PLWH’s abilities to restart and/or sustain ART or that, conversely, interfere with ART use. Primary among these was stable, high-quality housing. While the critical importance of housing for HIV health has been well described in the literature [80], the present study uncovers some of the critical mechanisms by which housing quality and stability support sustained ART use. First, as participants highlight, there is a stark difference between lower- and higher-quality housing. Lower-quality housing includes single-room occupancy residences, which, in many instances, are described as a hub of hassles, stress, and illegal activities, including drug selling, as well as lacking facilities needed for everyday life (e.g., kitchens and private bathrooms). Indeed, participants highlight that poor-quality housing is particularly risky for PLWH with past or current hazardous substance use, and indeed the vast majority of participants in the present study struggled with serious substance use problems, both past and present. Participants in lower-quality housing describe one common pathway to disruption of ART use, whereby peers in the setting trigger the PLWH’s drug use; this leads PLWH to “take a break” from ART, including under the (generally mistaken) assumption that individuals cannot benefit from ART when using substances [81]. In many cases, PLWH then begin to sell ART, mainly to predatory pharmacies, to pay for substances. Clearly, pharmacies offering to buy ART from PLWH, which is illegal, is a serious barrier to sustained ART [68, 69], since those with limited material resources will understandably find it challenging to decline extra funds. Commonly, the period off ART is lengthy, persisting until conditions change and/or PLWH are ready to reinitiate ART again. On the other hand, the understanding that one can take ART and use substances at the same time [70, 71] often helps PLWH sustain ART use even in the high-risk context of lower-quality housing, consistent with a harm reduction approach [82].

Further, unstable or substandard housing contributes to social isolation, which has a cascade of deleterious effects on the well-being and behavioral health of African American/Black and Latino PLWH, including regarding starting and sustaining ART. Based on the qualitative results, we found that social support and social engagement are fundamental to taking and sustaining ART, consistent with the past literature [83]. Further, also consistent with the past literature, we found substandard housing, moderate-to-high-risk levels of substance use, and stigma, which commonly prevented disclosure of HIV status to others, increase social isolation [84, 85]. The study extends this past literature by identifying a number of other factors that impede social support and contribute to social isolation. For example, loss of a close person such as friend or family member is common and precipitates ART discontinuation. Moreover, insufficient social support at times contributes to loss of the will to live, and “not caring” about oneself, which reduce motivation and ability to take ART, highlighting both the importance of social support and also how emotionally challenging it is to manage HIV over years, particularly for this subpopulation of PLWH. In addition, peer support and peer modeling are critical to restarting ART and effective ART management. Unexpectedly, social support was not related to the rate of starting and stopping ART in quantitative analyses, but a longer maximum duration of sustained ART, the more favorable outcome, was associated with lower levels of social support. We interpret this latter finding as highlighting the mixed effects of social support and the individuals who make up a social network. Indeed, as Lincoln [86] noted in a review article, research examining the relationship between social support and psychological well-being has largely ignored the negative side of social interactions. However, empirical evidence suggests that negative interactions can potentially be more harmful than social support is helpful [86]. For this subpopulation of PLWH, social network members may be in similarly financially constrained circumstances, and some may also be living with HIV [87]. Thus, the present study contributes to the literature on the complexities of the relationship between social support and psychological well-being, although more specificity on how social support and social connections might interfere with sustained ART use for this population is needed.

Qualitative and quantitative results highlight that substance use is one of the most common causes of ART discontinuation in this population. We identify a number of relationships and/or pathways between the initiation of substance use and subsequent ART discontinuation, in addition to the association with substandard housing described above. Qualitative results focus on substance use generally and do not unpack the effects of the different specific substances used. In quantitative results, we found cannabis use at a moderate-to-high-risk level, but not alcohol or other drugs, is associated with more stops and starts of ART regimens, and lifetime, but not recent, IDU is associated with fewer starts. Alcohol use at moderate-to-high-risk levels is associated with a shorter maximum duration of sustained ART, consistent with the past literature [88], and recent IDU is associated with a longer maximum duration of sustained ART. We discuss these findings on substance use in the following section.

Marijuana use is common among PLWH, who commonly use it for relaxation and social and therapeutic purposes, such as to alleviate anxiety and depression, improve appetite, relieve pain, and reduce HIV-related symptoms and ART side effects [89–91]. However, a recent Cochrane review noted a lack of data on the long-term effects of marijuana on morbidity and mortality among PLWH [92]. Past studies on the effects of marijuana use on ART uptake and adherence have shown mixed results. In some studies, decreased rates of adherence are found among marijuana users overall, but PLWH who use marijuana daily evidence high levels of adherence [90, 93]. Bonn-Miller and colleagues [89] found that heavy cannabis use, but not occasional use, is associated with lower ART adherence and greater HIV symptoms and ART side effects. The present study adds to the literature by highlighting that cannabis use, at moderate-to-high-risk levels, may exert an adverse influence on HIV-related behavior through its association with stopping and starting ART. Although the cross-sectional nature of the quantitative data does not allow us to determine causal pathways, mixed-methods findings discussed above suggest some of the mechanisms by which substance use, likely including marijuana use, may influence HIV-related behavior. As Bonn-Miller and colleagues [89] note, future prospective studies would elucidate malleable mechanisms that may underlie the observed relations, such as substance use motivation, emotion regulation, and cognitive functioning. Since cannabis use is highly prevalent among PWLH, harm reduction approaches may assist PLWH in keeping marijuana use at a non-hazardous level [73], which may support their abilities to sustain ART while using this substance.

We interpret the findings regarding the association of IDU with lower rates of starting and a longer duration on ART, both the more favorable outcomes, in the context of the lengthy periods that participants in the present study had lived with HIV and managed ART. Although there are often assumptions that IDU necessarily disrupts ART use [94], recent review articles and other studies highlight that with medication for opioid use disorder, appropriate supports, and easy access to medical care, persons who inject drugs can sustain ART with high levels of adherence [94, 95], although it can be challenging for PLWH who inject drugs to access these types of supportive services [96].

Participants highlight the emotional toll that living with HIV can take over a decade or more. In particular, the emotional toll of HIV management is often complicated by adverse contextual factors such as a loss of a loved one, social isolation, financial constraints, or poor-quality housing. They not uncommonly describe losing the will to live in these challenging contexts. Strengths and resilience are evident as well, as African American/Black and Latino PLWH commonly martial resources to restart ART again, but findings from the present study suggest that sustaining ART is always a challenge at some level, although it can be manageable. Earnshaw and colleagues [97] have proposed a “resilience agenda” for research that includes intervening on modifiable strength-based moderators of the association between societal stigma and disparities to reduce disparities. They argue for strengthening economic and community empowerment and trust at the structural level, creating common in-group identities and promoting contact with people living with HIV at the individual level, and enhancing social support and adaptive coping to improve resilience to societal stigma and ultimately reduce racial/ethnic HIV disparities. This resilience agenda is consistent with findings in the present study.

As noted above, we found concerns about stigma associated with HIV care and ART are associated with more stops and starts but not sustained ART. We interpret this in the context of the larger literature on stigma as a barrier to HIV care and ART [52]. Certainly, one cannot sustain ART if one does not initiate ART, and stigma interferes with the uptake of HIV medication [98]. Regarding mechanisms by which stigma may operate, our qualitative findings suggest that stigma and discrimination trigger medical distrust, which serves as a serious barrier to HIV care and ART, consistent with critical race theory [27]. On the other hand, stronger motivation for HIV care, a potentially modifiable factor, is associated with lower rates of both starting/stopping ART and sustaining ART. Although the present study did not focus on other forms of stigma, such as experiences related to substance use, sexual orientation, and gender identity, past studies suggest these are additional potent barriers to HIV care and ART use, including their intersectional effects [99]. Study findings certainly suggest that substance-use-related stigma is a potent force in the lives of this subpopulation of PLWH, creating direct and indirect barriers to engagement along the HIV care cascade.

### Limitations

The present study has limitations. First, HIV care and ART are continually evolving, including regarding treatment recommendations (e.g., when in the course of HIV disease to initiate ART) and the tolerability and efficacy of ART, which have improved dramatically over recent decades [100]. We interpreted findings in this context. However, the present study is cross-sectional and retrospective. We did not take a systematic life-history approach, and therefore the study likely lacks precision in the description of ART histories. Moreover, retrospective accounts such as these may be subject to primacy and recency effects, and other cognitive and memory biases [101–103]. On the other hand, accuracy of recall of traumatic events tends to be higher than for non-traumatic life events [104], and recall of life events using qualitative methods has validity [105]. While the study yields valuable findings, prospective longitudinal studies with real-time adherence monitoring and assessment of outcomes with biological markers are needed to identify the factors that promote or impede sustained ART use with more precision. Moreover, some of the measures used in the present study lack detail and may have therefore limited our ability to uncover associations. For example, qualitative results yielded rich descriptions of the role of housing quality and stability on ART use, but our single item on lifetime housing status was not associated with starting/stopping or sustaining ART at statistically significant levels. A more detailed and prospective assessment of housing that includes not just a lifetime incidence of homelessness but also housing stability, quality, and other features of housing, might yield quantitative results more similar to qualitative findings. Last, we elected to examine a wide range of variables at the bivariate level, and future studies to examine inter-relationships among risk and protective factors are needed. Nonetheless, the present study advances the literature on these under-studied phenomena in an under-studied population.

### Implications

Study findings have potential to inform, and potentially transform, clinical practice, social/behavioral interventions, policy, and future research. HIV care and research have focused mainly on ART adherence, as noted above. The present study highlights that stopping and starting ART are common for many PLWH in high-risk contexts and that stopping ART can serve as a barrier to HIV care. Thus, interventions to prevent ART discontinuation and reengage PLWH who have stopped ART have utility in clinical settings. For example, HIV care providers and behavioral scientists can attend to methods to predict ART stops, and clinical settings can regularly query patients about risk factors for stopping ART and also not assume someone on ART will sustain ART. These efforts can aim to prevent stopping ART, reduce the duration of ART stoppage, and maximize intervals of sustained ART use. In particular, younger PLWH and those newly diagnosed require support to initiate and sustain ART, and active outreach approaches to re-engage patients may be needed. Further, consistent with self-determination theory, study findings suggest that approaches that support PLWH’s autonomy, competence, and relatedness to build intrinsic motivation for ART, and that avoid pressuring African American/Black and Latino PLWH, may be particularly acceptable and efficacious in clinical practice. If living with HIV can be considered as a type of developmental process or “career,” findings suggest that different factors may influence ART use at various stages of the ART career. Our quantitative data do not allow the exploration of interactions with time, but future, prospective research could explore this. The present study highlights gaps in services for PLWH who use substances: results suggest stark differences between settings for persons who inject drugs (as IDU was associated with favorable outcomes) and settings that serve PLWH with substance use generally (since substance use was a serious barrier to taking and sustaining ART). Thus, settings for persons who inject drugs may have valuable insights to share with the larger community about treatment approaches that can be applied in other contexts. Further, the belief among some participants that substance use and ART use are incompatible is potentially modifiable. Regarding the larger context, clearly, it is illegal for pharmacies to offer to purchase ART from PLWH, and study findings highlight the important role that supportive, ethical pharmacies play in the lives of this population. Barriers such as poor-quality housing are addressable, and studies with similar populations have shown that providing quality housing can be effective [106] and cost-effective [107–109]. Last, the present study uncovers numerous ways that African American/Black and Latino PLWH in low-SES contexts successfully manage a range of serious challenges and thus demonstrate substantial resilience. Overall, the present study highlights the importance of a favorable social and structural context to foster long periods of sustained ART. Yet very often this favorable context is lacking. However, improving social/structural contextual environments and addressing psychosocial impediments to engagement along the HIV care continuum for this population is called for and would move the nation closer to ending the HIV epidemic.

## Conclusion

As Noysk and colleagues have noted [75], **“**ending the epidemic” will not happen in the United States without addressing racial/ethnic disparities. The present mixed-methods study advances the literature on long-term HIV survivorship, focusing on African American/Black and Latino PLWH from low-SES backgrounds, who have the greatest barriers to engagement along the HIV care continuum. Study findings will be of interest to public health leaders and other stakeholders who create policies to end the HIV epidemic and to HIV social service and medical providers generally.

## Supplementary Information

**Additional file 1:** Gwadz Heart to Heart2 Study Semi-Structured Interview Guide

## Data Availability

The datasets used and/or analysed during the current study are available from the corresponding author on reasonable request.

## Abbreviations

ACASI: Audio, computer-assisted self-interviewing
ART: Antiretroviral therapy (for HIV)
ACES-R: Adverse Childhood Experiences Scale-Revised
CAPI: Computer-assisted personal interviewing
CI: Confidence interval
HIV: Human immunodeficiency virus
IDU: Injection drug use
IRR: Incidence rate ratio
MOST: Multiphase optimization strategy
NYC: New York City
PLWH: People living with HIV
SES: Socioeconomic status
SU: Substance use
