# Supplementary material for: Stopping, starting, and sustaining HIV antiretroviral therapy: a mixed-methods exploration among African American/Black and Latino long-term survivors of HIV in an urban context
Source: BMC Public Health. 2021 Feb 27;21:419. doi: 10.1186/s12889-021-10464-x (PMC7912958; doi:10.1186/s12889-021-10464-x)
Supplement: Supplementary file 1 — Additional file 1: Gwadz Heart to Heart2 Study Semi-Structured Interview Guide [file 12889_2021_10464_MOESM1_ESM.docx]

**Heart to Heart 2 (HTH2) Study Semi-Structured Interview Guide**

**INTERVIEW 1**

WHAT: The main aim of the qualitative research effort is to add context and richness to participants’ experiences with intervention components, in order to inform future implementation of the optimized intervention. Should be focused on mechanisms of behavior change. Each component has a theoretical mechanism of action. Did the components actually “speak” to that mechanism of action? And if so, what other mechanisms of action were at play, if any? The component can have an effect on participants even if they do not initiate ART. These mechanisms are:

Component A, Sessions. Health beliefs (ART is necessary, concerns about ART), emotions (fear, distrust)

Component B, Pre-adherence preparation. Behavioral skills for adherence.

Component C. health coach/peer mentorship. Peer models and peer norms, social support and stigma

Component D. Focused support groups. Social support and stigma

Component E. Navigation. Structural barriers to health

It's all about their thinking about HIV care and ART and whether the intervention components had any effect on that, or any aspect of their life, and if anything changed for them in the intervention. And why and how. The understanding is that behavior change happens on their schedule, not ours, so we'll speak with them at 2 time periods in their engagement with HTH2.

Participants may or may not have initiated ART at the time of the QI. In either case, we want to understand their thinking (and feelings) about that, and whether their engagement in HTH2 had any effect on their thoughts, feelings, or actions.

We would also like to know how we could do this work better. What’s missing? What could be better?

Note that participants may give socially desirable answers. Reassure them we want to know what’s going on with them, and listen for changes in the “story.”

IN ADVANCE OF THE QI: The interviewer should know the participant’s condition arm and what components he/she was assigned to receive. Some arms have more activities than others, so we want to understand the experience of receiving relatively fewer components vs. receiving a great many components, in addition to participants’ take on each component. The participant may not know they components by name, so be prepared to describe it a little and use the name of the interventionist who delivered the component.

HOW: Interviews will be audio recorded and transcribed verbatim and transcripts will be analyzed. The interviews will last approximately 1 hour. Individuals will be compensated $25 for each qualitative interview. They will be informed about the compensation and time requirements in advance of the interview.

WHO: A small number of participants from each of the 16 conditions (2-4 participants/condition) will be randomly selected to engage in two qualitative interviews. The sample size will range from 32-64 participants, depending on our ability to locate participants and their response to the offer to engage in these activities.

WHEN: First interview toward the end of their intervention period, 2^nd^ interview between 3-6 months after intervention.

**AFTER THE INTERVIEW:**

The interviewers should keep in close contact with the research team, during the interview process, and share observations. Please take notes after the interviews to capture major concepts, observations, or themes and send them around to the qualitative research staff and Principal Investigator. Also please let us know if the guide works well or needs edits.

| **Domains of Inquiry** | **Sample questions and PROMPTS** |
| --- | --- |
| **INTRODUCTION1** | **Remind participant**   - **Interview is confidential** - **It’s OK if he/she is not engaged in HIV care or taking medication, we want to understand his/her experience** - **He/she can “pass” on any question he/she prefers not to answer** |
| **INTRODUCTION2** | **Tell me a little about yourself?** |
| Starting out: *Familiarize yourself with the person’s story (within reasonable limits).*  **Reasons for agreeing to participate in the intervention *now*.** Whether the response is compensation, “it was time,” friends recommended it, etc., still probe as to *why now*. This can be a good place for the participant to introduce themselves generally, but the interviewer should try and steer the interview *away* from rote HIV and/or substance use narratives and keep the interview relatively intervention-focused for now.  **Expectations vs. Reality.** If at this point the participant responds with only positive comparisons, gently remind her or him that we want to know about the not-so-good as well. Also, it is probably worth asking about *why* a participant had certain expectations and *whether* or *how* these were countered by their recent experiences with the HTH2. | **To start off, what was it that led you to agree to participate in HTH2?**  PROMPTS:   - Why at this particular point in your life/HIV trajectory? - Have you participated in anything similar in the past? And if so, how did that go?   **Is HTH2 what you expected? How or how not?**  PROMPTS:   - Had you heard anything about HTH2 prior to agreeing to participate? What specifically, and how did that make you feel (e.g. how did that influence your decision to participate, whether positive or negative)? - Before agreeing to participate, what did you think HTH2 would be like? Has that changed at all? How or how not? |
| **Experiences of Intervention.** General, non-guided impressions of intervention to this point. If participants have been involved in similar programs or interventions, ask whether this differs (broadly), then delve into specifics later. If individual components come up specifically, ask about them here and let this part of the interview develop organically (after all, the participant might not even differentiate between individual components. If not, follow up with the below. | **What do you think about HTH2 so far?**  PROMPTS:   - What stands out to you most about the HTH2 project so far? What have you like? Disliked? - Have you been involved in similar projects? How is HTH2 similar/different? - What do you think about the **staff** in general? (e.g. where they helpful, welcoming etc.?)   **So far, have any parts of HTH2 been particularly helpful to you?** **(DESCRIBE)**  **Has anything about HTH2 seemed particularly new or interesting**? **(DESCRIBE)** |
| ***NOTE: Intervention Components.*** *With knowledge of the person’s experimental condition [see table], probe for information about each component and its associated activities (counseling Interviewing sessions, pre-adherence preparation, support groups, peer mentorship, and patient navigation).*  *You might need to explain that difference activities are called “components.”* | ***SEE BELOW*** |
| **COMPONENT A** |  |
| **COMPONENT A: COUNSELING SESSIONS**  Component A, Sessions:  4 Individual bi-weekly sessions with a counselor who spoke with you about your relationship with health-care providers, and your feelings about taking HIV medication and helped unpack feelings about decision to take ART  -videos and handouts were used as well as conversations with Liz and/or Belkis  Interventionists: Liz, Belkis | **What do you remember about this component?**  What did you think about your individual sessions **counselor**?   - Can you tell me what kinds of things you discussed? - Had you ever talked about your feelings about medication and your provider before? What was that like for you in HTH2? - Can you describe the exercises in the sessions? Did you find them helpful? Surprising?   What do you think about the **videos**? (e.g. Did you find them useful?)   - Did you identify with them? How so?   **(IF NEEDED, ASK) Was this component useful to you? DESCRIBE**.  **How could this component/activity be improved?** |
| **COMPONENT B** |  |
| **COMPONENT B: PRE-ADHERENCE PREPARATION**  Component B, Pre-adherence preparation. Targets behavioral skills for adherence. 1 Individual session with a counselor where you discussed building an adherence habit with either HIV medication or other medication. In this component, you may have received some adherence tools such as a MEMS cap, pill box or app. Weekly phone check-ins for total of 6 weeks.  Interventionists: Mariam, Camille, Dawa | **What do you remember about this component?**  What did you think about **the pre-adherence preparation component**?   - Have the visual reminders been useful? Why or Why not? - Have the pill trial runs been helpful? Why or why not?   **(IF NEEDED, ASK) Was this component/activity useful to you? DESCRIBE**.  **How could this component/activity be improved?** |
| **COMPONENT C** |  |
| **COMPONENT C : PEER MENTOR/HEALTH COACH**  Component C. health coach/peer mentorship. Targets peer models and peer norms, social support and stigma. 4 Individual monthly sessions where you talked to a peer mentor about shared experiences around living with HIV and taking HIV medication.  HC: Donna, Daniel, Yvette, Shantae | **What do you remember about this component?**  What do you think abut your peer mentor/health coach?   - Can you tell me what kinds of things you discussed? - Did your peer mentor share his/her experiences with you? - Did you relate to what he/she shared? How so? - Was it helpful?   How often have you been in contact with your Peer Mentor **Interventionist**?   - By phone? In person? - Has he/she helped you out in any way (e.g. emotional support, guidance, referrals, etc.)?   **(IF NEEDED, ASK) Was this component/activity useful to you? DESCRIBE**.  **How could this component/activity be improved?** |
| **COMPONENT D** |  |
| **COMPONENT D : FOCUSED SUPPORT GROUPS**  Component D. Focused support groups. Social support and stigma. 6 bi-weekly support groups where you met with a group of 6-8 participants and discussed various issues pertaining to HIV and struggles with HIV medication, such as medical distrust, disclosure, etc.  Facilitator: KAREN  Might be Angie/Liz from earlier participants | **What do you remember about this component?**  What do you think about the **support groups**?  What kinds of things did you talk about? Can you give a specific example?  What has the group been like (i.e. describe the people in the group)? Could you identify with other group members and their experiences? How so?  How about the group leader?  Have you gotten anything out of the support groups? Please describe.  **(IF NEEDED, ASK) Was this component/activity useful to you? DESCRIBE**.  **How could this component/activity be improved?** |
| **GROUPS – what’s different about HTH2** | **Did the groups you attended at HTH2 seem different in any way from other groups you have attended?** (DESCRIBE) |
| **COMPONENT E** |  |
| **COMPONENT E : NAVIGATION**  Component E. Navigation. Targets structural barriers to health. Individual sessions with Nico for either 3 or 6 months. Sessions are both in person and by phone and could involve appointment accompaniment. Participants can attend navigation, as needed. Structural barriers (housing, referrals to health care providers, food resources) to health are addressed through referrals and using an intake and action plan.  Interventionist: Nico | **What do you remember about this component?**  What did you think about the **navigation activity**?   - What kind of services did she help you with? - Has your Navigation counselor help you get HIV care? - Has she/he helped you with referrals, communication with primary care provider, accompaniment to health care appointments, etc.?   How often have you been in contact with your Navigator?   - By phone? In person? - Has she helped you out in any way (e.g. emotional support, guidance, referrals, etc.)?Please describe   **Have you been able to get any other services as a result of the project?**  PROMPTS:  Housing, mental health, other physical health, substance use treatment?  **(IF NEEDED, ASK) Was this component/activity useful to you? DESCRIBE**.  **How could this component/activity be improved?** |
| **NAVIGATION: IF GOT HOUSING DURING NAVIGATION**  We would like to understand the effects of that, both positive and/or negative. Some people are getting housing in unfamiliar neighborhoods. How is that for them? | **(IF GOT HOUSING DURING HTH2 AS A DIRECT OR INDIRECT RESULT OF HTH2)**  **Tell us about your new housing situation. What has that been like for you?**  PROBE positive and negative aspects |
|  |  |
| **OVERALL** |  |
| **OVERALL DOSE** | **Overall, did you feel you had enough different activities with HTH2? Too many? Too few?** (EXPLORE) |

| **PART II** |  |
| --- | --- |
| **Thoughts about settings’ role in engagement in HIV care**  *Are people implicitly or explicitly discouraged from remaining in health care settings? Let’s get at this but not ask leading questions. The last question in this sub-section is more direct, while the other questions are more open-ended.* | **To what extent have you felt welcome and wanted at your HIV health care setting when you have been taking ART**? (EXPLORE)  **What about when you have not been taking ART**? (EXPLORE)  Our records show that – based on national guidelines – which you may not agree with, you were not attending health care appointments on the recommended schedule at the time you entered the HTH2 study. Why is that? (EXPLORE ASPECTS OF THE SETTING AND POLICIES OF THE SETTING AS WELL AS INDIVIDUAL FACTORS?)  (ASK IF NEEDED) Some people have felt unwelcome in HIV clinic settings, for example, when they were not taking ART, or when their viral load levels were detectable. Have you ever felt that way? (DESCRIBE, EXPLORE) |
| **Relationship to HIV CARE prior to agreeing to participate in HTH2.** | **Since you’ve been involved with HTH2, has anything changed about the way you think about HIV care**? (EXPLORE)  **Has anything changed about the way you feel about HIV care**? (EXPLORE)  **Have you attended HIV care appointments since you came to HTH2?** Why or why not? |
| **IF IT’S NOT CLEAR ALREADY, IS PARTICIPANT TAKING ART during HTH2?** | **Have you taken HIV medications since you joined HTH2? It’s OK if you haven’t. We just want to understand what’s going on with you now.**  Why or why not? (EXPLORE)  **What factors played a role in your deciding to take HIV medications at this time, whether related to HTH2 or other factors?**  **Are you still taking HIV medication?** (EXPLORE)  (IF YES) What has helped you stay on HIV medications?  (IF NO) Tell me about stopping taking HIV medications. (EXPLORE)  Do you plan to take HIV medications in the future? (EXPLORE) |
| **CHANGES DURING HTH2 RE: ART** | (IF NOT ALREADY COVERED) **Since you’ve been involved with HTH2, has anything changed about the way you think about HIV medication?**  **Has anything changed about the way you feel about HIV medication?**  PROMPTS:  [IF THIS HAS NOT ALREADY COME UP, EXPLORE] Let’s go back to when we were talking about the different parts of HTH2.  Are there any specific moments or activities you can remember that led you to have one of those “aha” moments where you began to think about or feel about HIV medications or HIV care differently? |
| **IF NOT TAKING ART AT PRESENT** | **Do you think that there is anything that would make you want to consider taking HIV medications on a regular basis?**  Why or why not? |
| **NOTE: KEEP THIS SECTION BRIEF**  **Relationship to ART prior to agreeing to participate in HTH2.** Hopefully this will have already come up by now, but if not, this might be a good place to ask about past ART experiences directly.  **HTH2’s influence on decisions regarding whether to (re)start ART.** Ask about whether and/or how one, a combination of several, or all of the components of the intervention, or even the intervention as a whole and larger than the sum of its parts, might have positively or negatively influenced a participant’s decision-making around (re)starting ART. Also the interviewer should leave open the possibility that some things having *negatively* influenced a participant’s thoughts regarding whether to (re)start ART might not necessarily conflict with anything that positively influenced these decisions. And finally, the interviewer should keep in mind that for the participant, a positive influence on their decisions re: ART might not necessarily mean that leads that person in the direction of (re)starting ART. | **Before you agreed to participate in HTH2 had you ever thought about taking HIV medication?**  PROMPTS:  Have you ever taken HIV medications in the past?   - IF YES: When did you start? How long did you take them? How regularly? Why did you stop? Can you tell me what else was going on in your life at the time? - IF NO: Can you tell me what else was going on in your life at the time?   Has anyone ever recommended that you take HIV medications?  IF YES: Do you remember how you reacted to that?  IF NO: Can you think of why they didn’t?  Have you ever felt pressured by anyone to take HIV medications in the past (e.g. HCP, friends, family, peers, society in general, etc.)?  IF YES: Can you tell me a little about that?  In the past, do you think that there is anything that has made it difficult for you to take HIV medications? Or that has made you not want to take them?  PROMPTS:  Substance use, medical distrust, side effects, stigma, etc. [IF THIS HAS NOT ALREADY COME UP REPEATEDLY, TRY TO REALLY GET SPECIFIC AT THIS POINT.] |
| **Acceptability, Feasibility, and Safety.**  Explore the acceptability of the intervention, including video components, exercises, participation of primary care providers, and other intervention content. Document the feasibility and safety (assessment of social impact or harms) of the intervention, including the number and length of sessions, format of the intervention, location and setting of the intervention, primary care provider involvement (if applicable) | **We talked about some of your experiences about HTH2, both positive and less positive. We want to ask a few more questions about how we can improve.**  **Has there been anything about HTH2 that you think has been particularly *unhelpful*?**  PROMPTS:   - Can you think of any negative experiences that you’ve had so far? - What do you think should be included in HTH2 that wasn’t included?   **Was it convenient for you to attend your sessions? Were there too many? Too few?**  **Is there anything about HTH2 so far that you thought was confusing?**  PROMPTS   - Has there been anything that you heard, any new information or anything that you thought could have been explained better? - Anything you’d like to have heard more about? - Has there been anything that didn’t make sense to you in terms of why it was part of HTH2 (e.g. why are we doing this)?   **Has there been anything so far that’s made it difficult for you to take part in HTH2?**  PROMPTS:  Confidentiality issues, intervention format and setting, transportation, costs, length of sessions, etc.  Is there anything you can think of that could have made your experience with the project better, more convenient, made it more comfortable for you to fully participate? |
|  |  |
| **Pressure/Distrust Related to HTH2.** There is an obvious socially desirable answer to questions in this part of the interview, especially in this particular circumstance, so even if there is hesitation on the part of the participant it is almost definitely worth probing a little further. | **I asked earlier if anyone has ever made you feel pressured to take HIV medication. Did you ever feel pressure during HTH2?** (DESCRIBE) |
| **SUBSTANCE USE TREATMENT EXPERIENCES** | *If a participant entered treatment or received support for substance use problems during HTH2, explore*  What kind of treatment/support?  Factors that prompted that decision  Aspects of the treatment that were helpful  Needs/gaps that remain  Relationships between changes in substance use patterns and ART decisions/uptake  Thoughts on abilities to maintain substance use goals and resources needed |
| **IF TIME ALLOWS**  INJECTABLE HIV MEDICATIONS: | EXPLAIN: In the near future there may be a new way of taking your HIV medications. Instead of taking pills every day, you could receive an injection under the skin at regular time periods that contains your HIV medicines. It could be a shot every month, or every two months.  You would not have to take pills.  You could come to the clinic to receive the injection, or administer it yourself.  People have a lot of reactions to this new way of taking HIV medication.  We want to know what you think.  What is your reaction to the idea of this new way of taking HIV medications?  (EXPLORE)  Do you think that would be something you would be interested in?  Why or what not?  (EXPLORE PERSPECTIVES ON PROS AND CONS AS APPROPRIATE)  Would you have any concerns about injectable HIV medication?  What could health care providers or HIV clinics to do help you decide about whether this new way of taking HIV medications would be right for you? |
| **CLOSING QUESTION** | **This is the last question. Is there anything I didn’t ask you about but that we should know about you and your experience in HTH2?** |
